# Protective role of curcumin in disease progression from non-alcoholic fatty liver disease to hepatocellular carcinoma: a meta-analysis

**DOI:** 10.3389/fphar.2024.1343193

**Published:** 2024-01-19

**Authors:** Yubing Li, Xinyu Deng, Xiyue Tan, Qianrong Li, Zhi Yu, Wenbin Wu, Xiao Ma, Jinhao Zeng, Xiaoyin Wang

**Affiliations:** ^1^ State Key Laboratory of Southwestern Chinese Medicine Resources, School of Pharmacy, Chengdu University of Traditional Chinese Medicine, Chengdu, China; ^2^ Department of Obstetrics, Hospital of Chengdu University of Traditional Chinese Medicine, Chengdu, China; ^3^ Health Care Office of the Service Bureau of Agency for Offices Administration of the Central Military Commission, Beijing, China; ^4^ TCM Regulating Metabolic Diseases Key Laboratory of Sichuan Province, Hospital of Chengdu University of Traditional Chinese Medicine, Chengdu, China

**Keywords:** curcumin, NAFLD, liver fibrosis, HCC, meta-analysis, preclinical

## Abstract

**Background:** Pathological progression from non-alcoholic fatty liver disease (NAFLD) to liver fibrosis (LF) to hepatocellular carcinoma (HCC) is a common dynamic state in many patients. Curcumin, a dietary supplement derived from the turmeric family, is expected to specifically inhibit the development of this progression. However, there is a lack of convincing evidence.

**Methods:** The studies published until June 2023 were searched in PubMed, Web of Science, Embase, and the Cochrane Library databases. The SYstematic Review Center for Laboratory animal Experimentation (SYRCLE) approach was used to evaluate the certainty of evidence. StataSE (version 15.1) and Origin 2021 software programs were used to analyze the critical indicators.

**Results:** Fifty-two studies involving 792 animals were included, and three disease models were reported. Curcumin demonstrates a significant improvement in key indicators across the stages of NAFLD, liver fibrosis, and HCC. We conducted a detailed analysis of common inflammatory markers IL-1β, IL-6, and TNF-α, which traverse the entire disease process. The research results reveal that curcumin effectively hinders disease progression at each stage by suppressing inflammation. Curcumin exerted hepatoprotective effects in the dose range from 100 to 400 mg/kg and treatment duration from 4 to 10 weeks. The mechanistic analysis reveals that curcumin primarily exerts its hepatoprotective effects by modulating multiple signaling pathways, including TLR4/NF-κB, Keap1/Nrf2, Bax/Bcl-2/Caspase 3, and TGF-β/Smad3.

**Conclusion:** In summary, curcumin has shown promising therapeutic effects during the overall progression of NAFLD–LF–HCC. It inhibited the pathological progression by synergistic mechanisms related to multiple pathways, including anti-inflammatory, antioxidant, and apoptosis regulation.

## Graphical Abstract

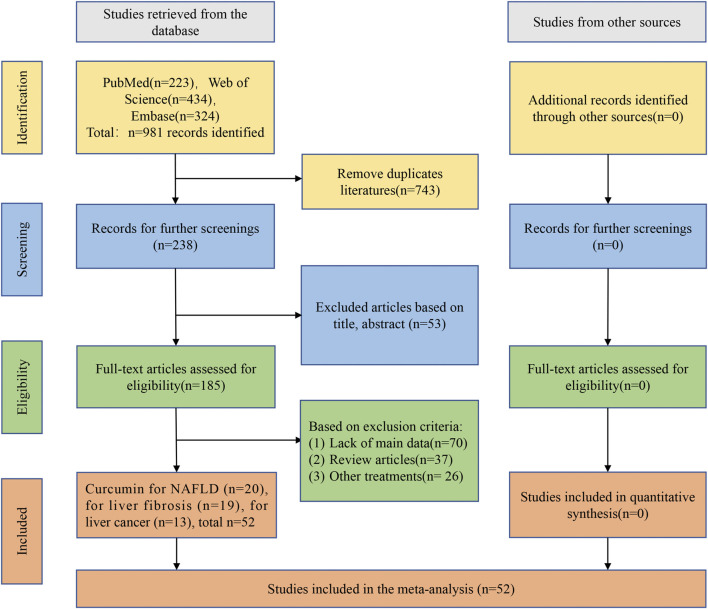

## Highlights

This is the first meta-analysis to correlate NAFLD, liver fibrosis, and hepatocellular carcinoma and evaluate the therapeutic effects of curcumin on systemic liver disease.
We explore the key mechanisms of curcumin for the treatment of systemic liver disease based on a mechanistic analysis combining these three diseases.
Curcumin inhibited the pathological progression by synergistic mechanisms related to multiple pathways, including anti-inflammatory, antioxidant, and apoptosis regulation.

## 1 Introduction

The progression from non-alcoholic fatty liver disease (NAFLD) to hepatocellular carcinoma (HCC) is a chronic and dynamic pathological process that involves complex factors and shows multiple pathological progressions. Non-alcoholic fatty liver has the potential to progress to non-alcoholic steatohepatitis (NASH), contributing to an augmented release of reactive oxygen species (ROS) and an inflammatory response. This, in turn, triggers hepatic fibrosis, characterized by the prolonged activation of hepatic stellate cells and extensive extracellular matrix (ECM) deposition, ultimately fostering the development of HCC. The multifaceted transformation process underscores the complexity of the disease’s progression. NAFLD stands out as the most prevalent chronic liver ailment, impacting approximately 25% of the global population (Anstee et al., 2019). Projections indicate a swift rise in the incidence of NAFLD in the coming decade (Marengo et al., 2016; Estes et al., 2018). As the predominant chronic liver condition globally, NAFLD exhibits critical manifestations, including chronic tissue damage, ROS elevation, inflammatory cytokines, and apoptotic factors during the NAFLD stage. These factors collectively activate hepatic stellate cells, thereby initiating the cascade toward liver fibrosis (LF) (Bruck et al., 2007; Wei et al., 2020). LF is a protective defense response to chronic liver injury. In recent years, the long-term nonintervention in the pathological process of NAFLD to NASH has been found to be a major causative factor for LF. Fibrosis is characterized by excessive collagen deposition between hepatocytes and hepatic sinusoids. Collagen is the most abundant protein in the extracellular matrix and is dominantly produced by hepatic stellate cells in the liver. At the same time, hepatic stellate cells inhibit the breakdown of ECM by producing metalloproteinases (Zhong et al., 2016). However, when the balance between the metalloproteinase synthesis and ECM degradation is disrupted, leading to the accumulation of ECM proteins in the liver, fibrosis can develop into cirrhosis (Algandaby et al., 2016). Cirrhosis is a major contributor to HCC, and studies have shown that in more than 80% of patients, cirrhosis will develop further into HCC (Bae et al., 2018). It is estimated that 23%–44% of patients suffering from NAFLD will develop NASH, leading to fibrosis in approximately 37%–41% of NASH sufferers, of which 10%–20% will develop cirrhosis (Sheka et al., 2020; Powell et al., 2021). It leads to HCC in 2.4%–12% of cirrhotic patients within 3–7 years (Moon et al., 2020). HCC is the terminal stage of chronic liver disease and the ultimate consequence of long-term untreated liver fibrosis, with a high mortality rate of 24%, which has become the leading public enemy of human health (Fattovich et al., 2004; Cao et al., 2015). Therefore, it is of great importance to prevent further progression of liver fibrosis and HCC in NAFLD patients. With the advanced understanding of the pathological process of NAFLD–LF–HCC by modern research, it exhibits dynamic changes. In particular, necrosis of liver parenchymal cells, leading to massive oxidative stress, excessive inflammatory response due to disruption of hepatic immune micro-homeostasis, and excessive proliferation of abnormal cells, contributes to the increasingly complex development of the disease. Therefore, systemic truncation of the pathological process targeting NAFLD–LF–HCC is an important strategy for the management of syndromic liver disease. However, with excellent anti-inflammatory, antioxidant, and anti-cancer properties, curcumin is expected to be an effective treatment for all stages of liver disease. In addition, how curcumin can effectively block the progression of this series of liver diseases needs to be urgently investigated.

Polyphenolic components comprise a major category of natural plant components, are widely distributed in turmeric, fruits, tea, and coffee, and have significant multi-signal modulating effects. Among them, curcumin is the most famous polyphenolic component, and modern research has shown that curcumin has excellent improving and regulating effects on a variety of liver diseases. Traditional Chinese and Indian medicine also use plants containing curcumin to treat a variety of clinical liver diseases (Hewlings and Kalman, 2017). In traditional Chinese medicine, curcumin is employed to address diverse liver conditions, drawing upon the hepatoprotective benefits documented in *XinXiu BenCao*, an ancient government-compiled work on traditional Chinese medicine in China. Similarly, in Indian traditional medicine, curcumin is utilized to treat a spectrum of liver diseases (Wright et al., 2013). Despite this historical application, there is currently no consolidated evidence to systematically confirm the effectiveness of curcumin across various liver diseases. Research on the association of nonalcoholic fatty liver disease progression to hepatocellular carcinoma is still lacking. We combined these three diseases to explore the critical mechanisms of curcumin in the treatment of systemic liver disease. What role does curcumin play in the series of hepatic diseases? How does it act in this pathological progression? These are two important questions waiting for answers. Therefore, this research applied meta-analysis combined with mechanism investigation to explore the systematic action of curcumin against NAFLD–LF–HCC. Then, it aims to clarify the action and mechanism characteristics of curcumin, and further provide evidence-based support for its systematic application.

## 2 Materials and methods

### 2.1 Data sources and search strategy

The studies focused on the hepatoprotective effect of curcumin in animals published until June 2023 were searched in PubMed, Web of Science, Embase, and the Cochrane Library databases. The search term is identified as the treatment combined with the disease and specific search strategies such as participants, intervention, comparison, outcome, and study design. The search term for treatment is “curcumin.” The disease search terms are “non-alcoholic fatty liver disease,” “NAFLD,” “NASH,” “liver fibrosis,” “liver cancer,” “HCC,” and “hepatocellular carcinoma” (Supplementary Table S1).

### 2.2 Eligibility criteria

The criteria for including the preclinical literature are as follows: 1) Population species and study type: rodent to establish NAFLD, LF, and HCC models; 2) interventions and comparison: the experimental group was only administered curcumin at a certain dose, whereas the model group was administered equal amounts of saline or distilled water by the same route.; 3) outcome indicators: for curcumin treatment for NAFLD, the primary outcome measures included liver function and lipid metabolism. Other indices were selected for secondary outcome measures. For curcumin treatment for LF, the primary outcome measures included liver function, collagen fiber levels, and the marker of hepatic stellate cell activation. Other indices were selected for secondary outcome measures. For curcumin treatment for HCC, the primary outcome measures included tumor volume and weight.

Exclusion criteria for the preclinical literature are as follows: 1) population species and study type not belonging to NAFLD, LF, or HCC models and other types of articles such as reviews, editorials, and meta-analysis; 2) intervention: the experimental group did not use curcumin or other drugs besides curcumin; 3) comparison: no control group corresponding to the curcumin group; 4) outcome: absence of critical outcome indicators and lack of data.

### 2.3 Data extraction

Two independent authors evaluated the remaining studies based on the inclusion and exclusion criteria after eliminating duplicates. An initial review of the titles and abstracts was then conducted, and the full text of the relevant studies was reviewed to assess their applicability according to the PRISMA criteria (Moher et al., 2009). All the following disagreements were resolved by consensus: 1) the year of publication and the first author; 2) the approaches to induce animal disease models and the modeling standard; 3) the regimen features described in the intervention and comparison as dosage, frequency or the period of administration, delivery route, and vehicle; 4) characteristics of different types of experiments that were extracted based on the age, gender, sample size, model type, and animal species; and 5) alterations in the levels of disease indicators pre- and post-treatment. For some literature studies that needed to extract data from images, we extracted the corresponding outcome indicators from graphs using digital ruler software.

### 2.4 Risk of bias and the quality of evidence assessment

Two researchers independently assessed the quality of the included studies. The SYstematic Review Center for Laboratory animal Experimentation (SYRCLE) risk of a bias assessment tool for preclinical studies was used (Hooijmans et al., 2014). The SYRCLE tool consists of 10 items that were all included in the evaluation’s contents. Moreover, the final evaluation result is the low risk of bias or high risk of bias, and the literature quality evaluation includes 10 items, as shown in Figure 2.

### 2.5 Statistical analysis

StataSE software (version 15.1) was used for all analyses. The random-effect model was used to pool the results after considering the various animal types and experimental designs. The effect sizes were represented using the standard mean difference (SMD) and 95% confidence interval (95% CI). Heterogeneity was evaluated by I-square (*I* ^*2*^). When *I* ^*2*^ < 50%, it indicates good heterogeneity in the included studies. When *I* ^*2*^ > 50%, it implies that there is heterogeneity in the included studies. We used *P* as the statistical difference, and when *p* < 0.05, it was regarded as considerably significant. In addition, publication bias was evaluated using Egger’s test, with |t| < 0.05 defined as a significant publication bias. Specifically, dose-duration analysis was conducted to determine the preclinical optimal duration and dosage by Origin 2021 software.

### 2.6 Time–dosage analysis

Origin 2021 software was used to generate three-dimensional (3D) images to determine the best administration time and dose of dosage based on the statistical significance of the included data.

### 2.7 Mechanism analysis

Fifty-two articles included in this meta-analysis were summarized in mechanistic pathways or targets. In addition, the relevant literature was consulted to refine the specific mechanisms of disease progression in NAFLD–LF–HCC.

## 3 Result

### 3.1 Screening process for the included literature

A total of 981 articles were initially retrieved, comprising 223 from PubMed, 434 from Web of Science, and 324 from Embase. After eliminating 743 duplicates, 238 articles remained for subsequent screening. Upon reviewing titles and abstracts, 53 articles were excluded. The remaining 185 items underwent further scrutiny. Among them, 70 lacked essential data, 37 were review articles, and 26 involved treatments other than curcumin. Ultimately, a total of 52 preclinical studies met the inclusion criteria for further analyses, including 20 studies that reported curcumin treatment for NAFLD, 19 studies that reported curcumin for LF, and 13 studies that reported curcumin for HCC (graphical abstract).

### 3.2 Characteristics of studies included in the analysis

**FIGURE 1:**
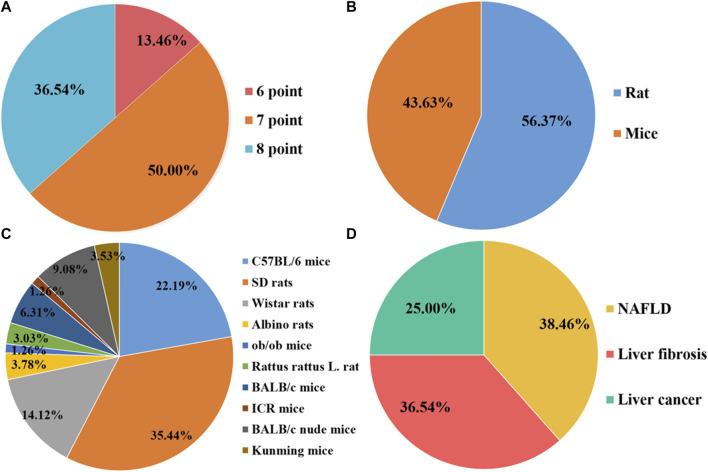
Characteristics of the eligible studies. **(A)** Quality assessment scores; **(B)** Rats/Mice; **(C)** Animal species; **(D)** Disease category.

A total of 20 preclinical studies reported curcumin treatment for NAFLD with a total of 330 animals. The animal species involved were Sprague–Dawley rats, C57BL/6 mice, Albino rats, and C57BL/6J ob/ob mice. A total of 15 studies (Cunningham et al., 2018; Ding et al., 2018; Li et al., 2018; Yan et al., 2018; Mahmoud et al., 2018; Feng et al., 2018; Feng et al., 2019; Gheibi et al., 2019; Lee et al., 2020; Li et al., 2021; Liu et al., 2017; Abd El-Hameed et al., 2021; Sun et al., 2021; Chen et al., 2022; Abd‐Elrazek et al., 2022) reported that a high-fat diet (HFD) model was established, 4 studies (Wan et al., 2014; Kim et al., 2016; Tong et al., 2021; Lee et al., 2022) used a methionine–choline-deficient diet (MCD) model, and only 1 study (Kuo et al., 2012) used C57BL/6J ob/ob mice as an obesity model (Figure 1). The duration of the administration varied from 2 weeks to 16 weeks, and the dosage ranged from 50 mg/kg to 200 mg/kg. The animals in the experimental group were all treated with curcumin based on the NAFLD model. The critical prognostic indicators consist of liver function indicators such as alanine aminotransferase (ALT), aspartate transaminase (AST), and liver fat indexes such as triglyceride (TG), total cholesterol (TC), high-density lipoprotein cholesterol (HDL-C), and low-density lipoprotein cholesterol (LDL-C). Some other pharmacological indexes, such as serum insulin, glucose, and homeostasis model assessment-insulin resistance (HOMA-IR), were also recorded. In addition, the indicators related to oxidative stress and inflammation containing superoxide dismutase (SOD), malondialdehyde (MDA), glutathione (GSH), interleukin-6 (IL-6), interleukin-1β (IL-1β), and tumor necrosis factor-α (TNF-α) were also included (Table 1).

**TABLE 1:** Characteristics of the 52 included studies.

Author(s)/year	Disease category	Species	Model	Sample size (n)	Drug dosage and model method	Treatment courses	Main outcome indicators
				Cur/model			
Lee et al. (2022)	NAFLD	C57BL/6 mice	MCD	10 (5/5)	Cur:Cur 120 mg/kg/d +MCD diet Mod:MCD diet	4 weeks	TG, TC, LDL, HDL, IL-6, TNF-α, and glucose
Chen et al. (2022)	NAFLD	SD rats	HFD	16 (8/8)	Cur:Cur 6 mg/kg/d + HFD diet	4 weeks	TG, TC, ALT, AST, LDL, HDL, insulin, glucose, HOMA-IR, and liver index
					Mod:HFD + streptozotocin		
Abd‐Elrazek et al. (2022)	NAFLD	SD rats	HFD	12 (6/6)	Cur:Cur 200 mg/kg/d + HFD diet	4 weeks	TG, TC, HDL, insulin, MDA, GSH, and liver index
					Mod:HFD diet		
Abd El-Hameed et al. (2021)	NAFLD	SD rats	HFD	20 (10/10)	Cur:Cur 50 mg/kg/d + HFD diet	2 weeks	ALT, AST, and liver index
					Mod:HFD diet		
Tong et al. (2021)	NAFLD	C57BL/6 mice	MCD	16 (8/8)	Cur:Cur 100 mg/kg/d + HFD diet	8 weeks	ALT, AST, TNF-α, IL-1β, and glucose
					Mod:HFD diet		
Sun et al. (2021)	NAFLD	C57BL/6 mice	HFD	16 (8/8)	Cur:Cur 150 mg/kg/d + HFD diet	6 weeks	TG, TC, ALT, AST, LDL, HDL, insulin, glucose, HOMA-IR, and liver index
					Mod:HFD diet		
Li et al. (2021)	NAFLD	SD rats	HFD	14 (7/7)	Cur:Cur 200 mg/kg/d + HFD diet	4 weeks	TG, TC, ALT, AST, LDL, HDL, IL-6, TNF-α, and IL-1β
					Mod:HFD diet		
Lee et al. (2020)	NAFLD	C57BL/6 mice	HFD	20 (10/10)	Cur:Cur 100 mg/kg/d + HFD diet	13 weeks	TG, ALT, and AST
					Mod:HFD diet		
Feng et al. (2019)	NAFLD	C57BL/6 mice	HFD	20 (10/10)	Cur:Cur 0.1% w/w + HFD diet	16 weeks	TG, TC, ALT, AST, LDL, HDL, TNF-α, and IL-1β
					Mod:HFD diet		
Gheibi et al. (2019)	NAFLD	Wistar rats	HFD	12 (6/6)	Cur:Cur 200 mg/kg/d + HFD diet Mod:HFD diet	4 weeks	TG, LDL, HDL, SOD, and MDA
Yan et al. (2018)	NAFLD	C57BL/6 mice	HFD	12 (6/6)	Cur:Cur 100 mg/kg/d + HFD diet	4 weeks	TG, TC, ALT, insulin, glucose, HOMA-IR, and liver index
					Mod:HFD diet		
Cunningham et al. (2018)	NAFLD	SD rats	HFD	24 (12/12)	Cur:Cur 100 mg/kg/d + HFD diet	12 weeks	ALT, AST, SOD, GSH, TNF-α, and insulin
					Mod:HFD diet		
Li et al. (2018)	NAFLD	SD rats	HFD	17 (10/7)	Cur:Cur 80 mg/kg/d + HFD diet	5 weeks	TG, TC, ALT, insulin, and glucose
					Mod:HFD diet		
Ding et al. (2018)	NAFLD	SD rats	HFD	12 (6/6)	Cur:Cur 60 mg/kg/d + HFD diet	6 weeks	TG, TC, LDL, and IL-1β
					Mod:HFD diet		
Feng et al. (2019)	NAFLD	SD rats	HFD	20 (10/10)	Cur:Cur 100 mg/kg/d + HFD diet	8 weeks	TG, TC, ALT, AST, LDL, HDL, FFA, MDA, GSH, and TNF-α
					Mod:HFD diet		
Mahmoud et al. (2018)	NAFLD	Albino rats	HFD	30 (15/15)	Cur:4 g/kg/d Cur diet + HFD diet	12 weeks	ALT, AST, IL-6, and TNF-α
					Mod:HFD diet		
Liu et al. (2017)	NAFLD	C57BL/6 mice	HFD	20 (10/10)	Cur:Cur 100 mg/kg/d + HFD diet	16 weeks	TG, TC, ALT, AST, LDL, HDL, and FFA
					Mod:HFD diet		
Kim et al. (2016)	NAFLD	C57BL/6 mice	MCD	14 (7/7)	Cur:Cur 100 mg/kg/d +MCD diet	4 weeks	TG, TC, ALT, AST, LDL, HDL, IL-6, and TNF-α
					Mod:MCD diet		
Wang et al. (2012)	NAFLD	C57BL/6 mice	MCD	16 (8/8)	Cur:Cur 2% w/w + MCD diet	2 weeks	TG, TC, LDL, HDL, IL-6, and TNF-α
					Mod:MCD diet		
Kuo et al. (2012)	NAFLD	C57BL/6J ob/ob mice	ob/ob mice	10 (5/5)	Cur:Cur 3% w/w + HFD diet	8 weeks	TG, TC, ALT, GSH, IL-6, and TNF-α
					Mod:HFD diet		
Elswefy et al. (2020)	Liver fibrosis	Wistar rats	Liver fibrosis	20 (10/10)	Cur:Cur 100 mg/kg/d	8 weeks	ALT, AST, area of collagen fibrosis in the liver (%), hydroxyproline, MDA, and GSH
					Mod:BPA 50 mg/kg/day for 8 w		
Gowifel et al. (2020)	Liver fibrosis	Wistar rats	TAA	16 (8/8)	Cur:Cur 200 mg/kg/d	8 weeks	ALT, AST, ALP, collagen level, α-SMA, MDA, GSH, TNF-α, and TGF-β
					Mod:injected TAA 100 mg/kg, i.p. three times a week, for 8 weeks		
Kong et al. (2020)	Liver fibrosis	SD rats	CCl_4_	12 (6/6)	Cur:Cur 400 mg/kg once 2 days	8 weeks	ALT, AST, ALP, collagen level, α-SMA, hyaluronidase (HA), PCⅢ, and hydroxyproline
					Mod:50% olive oil CCl_4_ was		
					injected 1 mL/kg, twice a week		
Hernández-Aquino et al. (2020)	Liver fibrosis	Wistar rats	CCl_4_	16 (8/8)	Cur:Cur 100 mg/kg twice a day	4 weeks	ALT, collagen level, α-SMA, GSH, TGF-β, and IL-1β
					Mod:400 mg/kg CCl_4_ oil i.p. three times per week, for 12 weeks		
Khodarahmi et al. (2020)	Liver fibrosis	Wistar rats	BDL	16 (8/8)	Cur:Cur 100 mg/kg/day	4 weeks	ALT, AST, ALP, collagen level, MDA, and TGF-β
					Mod:BDL induced liver fibrosis		
Abo-Zaid et al. (2020)	Liver fibrosis	*Rattus rattus* L. rats	CCl_4_	24 (12/12)	Cur:Cur 250 mg/kg/day	6 weeks	ALT, AST, TNF-α, and TGF-β
					Mod:injected 3 mL/kg CCl_4_ two times weekly for 6 weeks		
Eshaghian et al. (2018)	Liver fibrosis	Wistar rats	BDL	16 (8/8)	Cur:Cur 100 mg/kg/day	4 weeks	ALT, AST, ALP, collagen level, α-SMA, MDA TNF-α, TGF-β, and IL-1β
					Mod:BDL induced liver fibrosis		
Qin et al. (2018)	Liver fibrosis	SD rats	CCl_4_	20 (10/10)	Cur:Cur 200 mg/kg/day	8 weeks	ALT, AST, the area of collagen fibrosis in the liver (%), α-SMA, hyaluronidase (HA), laminin (LN), and PCⅢ
					Mod:injected 1.5 mL/kg CCl_4_ oil every 3 days for 8 weeks		
Zhao et al. (2018)	Liver fibrosis	C57BL/6 mice	CCl_4_	20 (10/10)	Cur:Cur 200 mg/kg/day	4 weeks	ALT, AST, collagen level, the area of collagen fibrosis in the liver (%), α-SMA, TNF-α, TGF-β, IL-6, and IL-1β
					Mod:injected 3 μL/g CCl_4_ oil twice weekly for 4 weeks		
Jin et al. (2016)	Liver fibrosis	SD rats	CCl_4_	12 (6/6)	Cur:Cur 300 mg/kg/day	4 weeks	ALT, AST, hyaluronidase (HA), laminin (LN), PCⅢ, and hydroxyproline
					Mod:injected 0.1 mL/100 g body weight CCl_4_ oil		
Huang et al. (2016)	Liver fibrosis	C57BL/6 mice	CCl_4_	12 (6/6)	Cur:Cur 100 mg/kg/day	12 weeks	ALT, AST, collagen level, α-SMA, hydroxyproline, TNF-α, TGF-β, and IL-6
					Mod:injected 0.1 mL/100 g body weight CCl_4_ oil twice a week		
Wu et al. (2016)	Liver fibrosis	BALB/c mice	CCl_4_	12 (6/6)	Cur:Cur 200 mg/kg, three times a week	8 weeks	ALT, AST, collagen level, and α-SMA
					Mod:injected 1 mL/kg body weight CCl_4_ oil twice a week for 8 weeks		
Zhang et al. (2012)	Liver fibrosis	SD rats	CCl_4_	20 (10/10)	Cur:Cur 200 mg/kg/d	6 weeks	ALT, AST, ALP, collagen level, α-SMA, hyaluronidase (HA), laminin (LN), and PCⅢ
					Mod:injected 1 mL/kg body weight CCl_4_ oil two times every remaining week for 6 weeks		
El Swefy et al. (2016)	Liver fibrosis	Wistar rats	BDL	16 (8/8)	Cur:Cur 20 mg/kg/d + BDL	2 weeks	ALT, AST, the area of collagen fibrosis in the liver (%), hydroxyproline, MDA, and GSH
					Mod:Bile duct-ligated + CMC		
Zhao et al. (2014)	Liver fibrosis	SD rats	CCl_4_	20 (10/10)	Cur:Cur 400 mg/kg/d	6 weeks	ALT, AST, ALP, collagen level, and α-SMA
					Mod:injected 300 μL/100 g CCl_4_ oil twice a week over a period of 6 weeks		
Wang et al. (2012)	Liver fibrosis	BALB/c mice	TAA	12 (6/6)	Cur:Cur 300 mg/kg, three times a week	8 weeks	ALT, AST, collagen level, the area of collagen fibrosis in the liver (%), and TNF-α
					Mod:injected TAA 100 mg/kg three times a week for 8 weeks		
Yao et al. (2012)	Liver fibrosis	SD rats	CCl_4_	30 (15/15)	Cur:Cur 200 mg/kg/d	6 weeks	Collagen level, the area of collagen fibrosis in the liver (%), α-SMA, and TGF-β
					Mod:injected 2 mL/kg body weight CCl_4_ oil twice weekly for up to 6 weeks		
Bassiouny et al. (2011)	Liver fibrosis	SD rats	CCl_4_	20 (10/10)	Cur:Cur 200 mg/kg/d	8 weeks	ALT, AST, MDA, GSH, TNF-α, TGF-β, and IL-6
					Mod:injected 0.3 mL/kg body weight CCl_4_ oil once per week for 4 weeks, followed by injecting twice weekly for 12 weeks		
Fu et al. (2008)	Liver fibrosis	SD rats	CCl_4_	12 (6/6)	Cur:Cur 400 mg/kg/d	8 weeks	ALT, AST, ALP, collagen level, α-SMA, hydroxyproline, GSH, TNF-α, and IL-6
					Mod:injected 1 mL/kg body weight CCl_4_ oil every other day for 8 weeks		
Zeng et al. (2022)	Liver cancer	BALB/C nude mice	Xenograft tumors	10 (5/5)	Cur:5 mg/kg every 3 days, six injections, intravenously	18 days	Tumor volume and weight Tumor volume = 1/2 (length× width ^2)
					Mod:BALB/C nude mice of HepG2 xenograft tumors when the tumor volume reached 100 mm^3^		
Wei et al. (2022)	Liver cancer	ICR mice	Xenograft tumors	10 (5/5)	Cur:100 mg/Kg/d intragastrical administration Mod:Hep-G2 cells were inoculated subcutaneously into the right axillary of each mouse	12 days	Tumor volume and weight Tumor volume = 1/6×π×length ×width ^2
Qi et al. (2021)	Liver cancer	BALB/c mice	Xenograft tumors	8 (4/4)	Cur:5 mg/kg every 2 days, seven injections, intravenously	14 days	Tumor volume Tumor volume = 1/2 (length× width ^2)
					Mod:H22 cells were injected into the right flank of female BALB/c mice		
Khan et al. (2021)	Liver cancer	Nude mice	Xenograft tumors	10 (5/5)	Cur:50 mg/kg/d intraperitoneal injection	21 days	Tumor volume and weight Tumor volume = 1/2 (length× width ^2)
					Mod:Hep3B cells were injected subcutaneously into the right flank of male nude mice		
Tian et al. (2021)	Liver cancer	BALB/c nude mice	Xenograft tumors	14 (7/7)	Cur:240 mg/kg/d intragastric administration	14 days	Tumor volume and weight Tumor volume = 1/2 (length× width ^2)
					Mod:HepG2 cells in 0.1 mL saline were injected into the right flank from the back of the mice		
Wang et al. (2021)	Liver cancer	Kunming mice	Xenograft tumors	6 (3/3)	Cur:9.9 mg/kg/d intravenous administration; Mod:H22 cells were subcutaneously injected at the right forelimb axilla	7 days	Tumor volume and weight Tumor volume = 1/2 (length× width ^2)
Guo et al. (2021)	Liver cancer	BALB/c mice	Xenograft tumors	10 (5/5)	Cur:free curcumin	15 days	Tumor volume
					Mod:HepG2 tumor cells were subcutaneously injected		Tumor volume = 1/2 (length× width ^2)
Kianamiri et al. (2020)	Liver cancer	BALB/c mice	Xenograft tumors	8 (4/4)	Cur:20 mg/kg/d intraperitoneal administration	24 days	Tumor volume
					Mod:Hepa1-6 cells were subcutaneously injected into the flank of the mice		
Deng et al. (2020)	Liver cancer	BALB/c-nu/nu mice	Xenograft tumors	14 (7/7)	Cur:200 mg/kg/d intragastrical administration	21 days	Tumor volume
					Mod:Hepa1-6 cells were subcutaneously injected into the flank of the mice		Tumor volume = 1/2 (length× width ^2)
Wang et al. (2019)	Liver cancer	BALB/C- nude mice	Xenograft tumors	12 (6/6)	Cur:20 mg/kg/d intraperitoneal administration; Mod:HCCLM3 cells were subcutaneously injected		Tumor volume
							Tumor volume = length × width × height/2
Chen et al. (2019)	Liver cancer	Kunming strain mice	Xenograft tumors	12 (6/6)	Cur:10 mg/kg/d intravenous administration; Mod:HCCLM3 cells were subcutaneously injected	7 days	Tumor volume
							Tumor volume = 1/2 (length× width ^2)
Cheng et al. (2018)	Liver cancer	Kunming mice	Xenograft tumors	10 (5/5)	Cur:9.8 mg/kg intravenously injected every 2 days for a total of four injections	8 days	Tumor volume Tumor volume = length × width ^2 × 0.52
					Mod:hepatoma H22 xenograft tumor-bearing Kunming mice		
Xing et al. (2018)	Liver cancer	Nude mice	Xenograft tumors	12 (6/6)	Cur:5 mg/kg intravenously administered every 3 days for a total of seven injections	23 days	Tumor volume and weight
					Mod:HepG2 xenograft tumor-bearing nude mouse models were replicated		Tumor volume = length × width ^2 × 0.52

A total of 19 preclinical studies reported curcumin treatment for LF and contained a total of 326 animals. The animal species involved were Sprague–Dawley rats, C57BL/6 mice, BALB/c mice, and Wistar rats. A total of 13 studies (Kong et al., 2020; Hernández-Aquino et al., 2020; Abo-Zaid et al., 2020; Qin et al., 2018; Zhao et al., 2018; Jin et al., 2016; Huang et al., 2016; Wu et al., 2016; Zhang et al., 2012; Zhao et al., 2014; Yao et al., 2012) reported that carbon tetrachloride (CCl_4_)-induced LF model was established, 3 studies (El Swefy et al., 2016; Eshaghian et al., 2018; Khodarahmi et al., 2020) used bile duct ligation to induce a liver fibrosis model, 2 studies (Wang et al., 2012; Gowifel et al., 2020) used thioacetamide (TAA) to induce a liver fibrosis model, and only 1 study (Elswefy et al., 2020) used gavage with bisphenol A (BPA) to induce liver fibrosis. The duration of the administration varied from 2 weeks to 12 weeks, and the dosage ranged from 20 mg/kg to 400 mg/kg. The animals in the experimental group were all treated with curcumin based on the liver fibrosis model. The main outcome indicators included liver function indexes, such as ALT, AST, and alkaline phosphatase (ALP), and liver fibrosis indexes, such as collagen, area of collagen fibrosis in the liver (%), α-smooth muscle actin (α-SMA), hyaluronidase (HA), laminin (LN), precollagen type Ⅲ (PCⅢ), and hydroxyproline (Hyp). MDA and GSH were also recorded. In addition, the indicators related to inflammation containing IL-6, IL-1β, transforming growth factor-β (TGF-β), and TNF-α were also included (Table 1).

A total of 13 preclinical studies investigating curcumin treatment for HCC included a total of 136 animals, encompassing BALB/c nude mice, ICR mice, and Kunming mice. All 13 studies utilized xenograft tumors to establish the HCC model. Among these, five studies (Cheng et al., 2018; Chen et al., 2019; Qi et al., 2021; Wang et al., 2021; Zeng et al., 2022) reported curcumin administration via tail intravenous injection, four studies (Xing et al., 2018; Wang et al., 2019; Kianamiri et al., 2020; Khan et al., 2021) via intraperitoneal injection, and three studies (Deng et al., 2020; Tian et al., 2021; Wei et al., 2022) via gavage. Only one study (Guo et al., 2021) did not specify the administration method. Administration duration ranged from 7 to 24 days, and dosages varied from 5 mg/kg to 240 mg/kg based on the administration method. In all the experimental groups, the animals received curcumin treatment in conjunction with the HCC model. Key outcome indicators included tumor volume and weight (Table 1).

### 3.3 Quality evaluation

**FIGURE 2:**
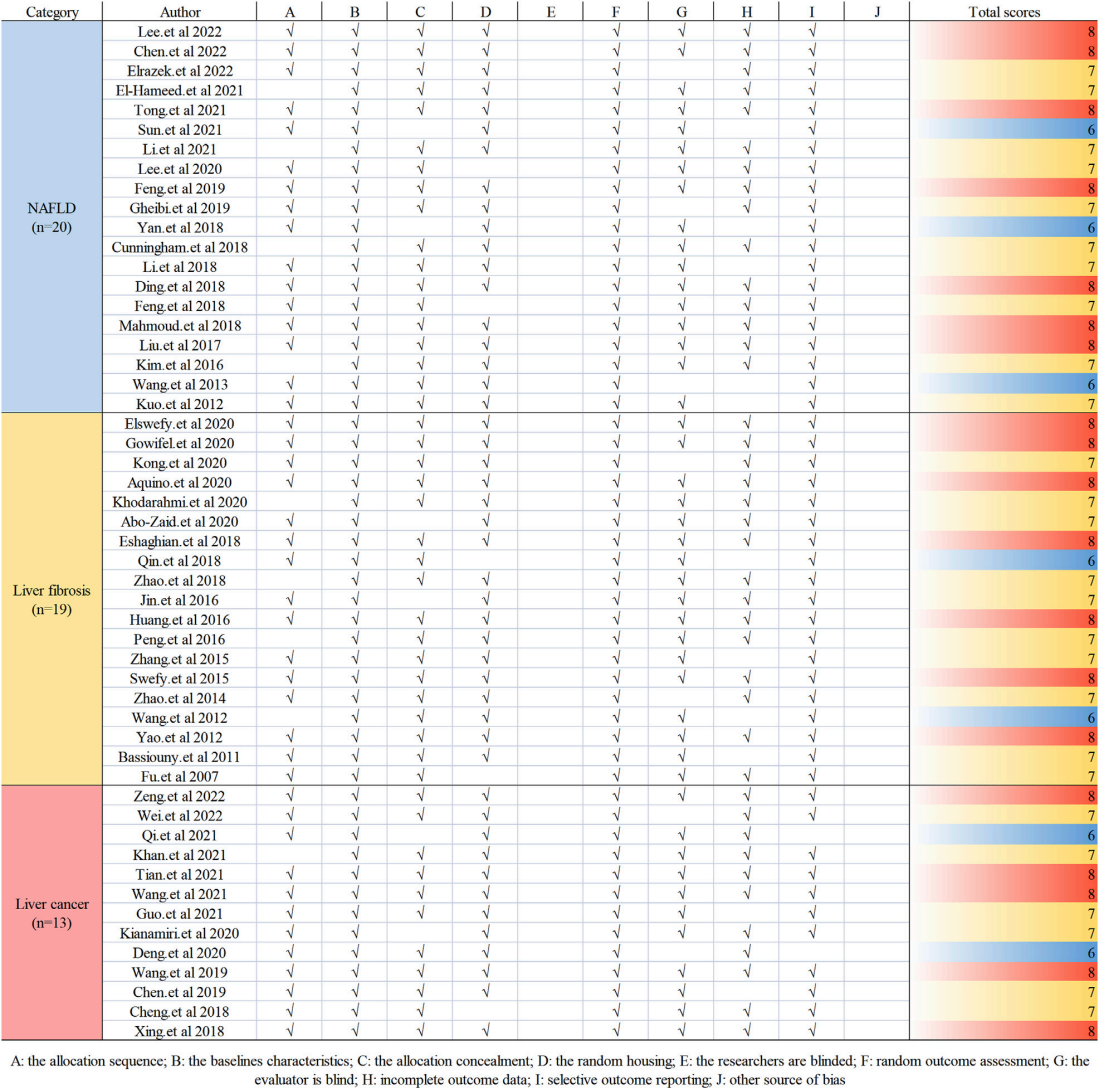
Risk-of-bias and quality assessment scores for each article.

Overall, the quality scores of the selected publications ranged from 6 to 8, according to the SYRCLE 10-item checklist, with a mean score of 7.23 (out of 10). Of the 52 items listed, 7 items scored 6 (13.46%), 26 items scored 7 (36.54%), and 19 items scored 8 (50.00%). However, none of the studies mentioned blinding of the investigators, and none reported the presence of other biases. The methodological quality of each survey is summarized in Figure 2.

### 3.4 Effect of curcumin on NAFLD

A meta-analysis of the included literature on curcumin for NAFLD was performed. Indicators of liver function, lipid metabolism, oxidative stress, and inflammation were assessed separately, and the potential tendency of curcumin on these indicators was explored. The results suggest that curcumin may improve NAFLD by effectively affecting liver function indices and lipid metabolism levels through anti-inflammatory and anti-oxidative stress pathways.

#### 3.4.1 The effect on liver function

**FIGURE 3:**
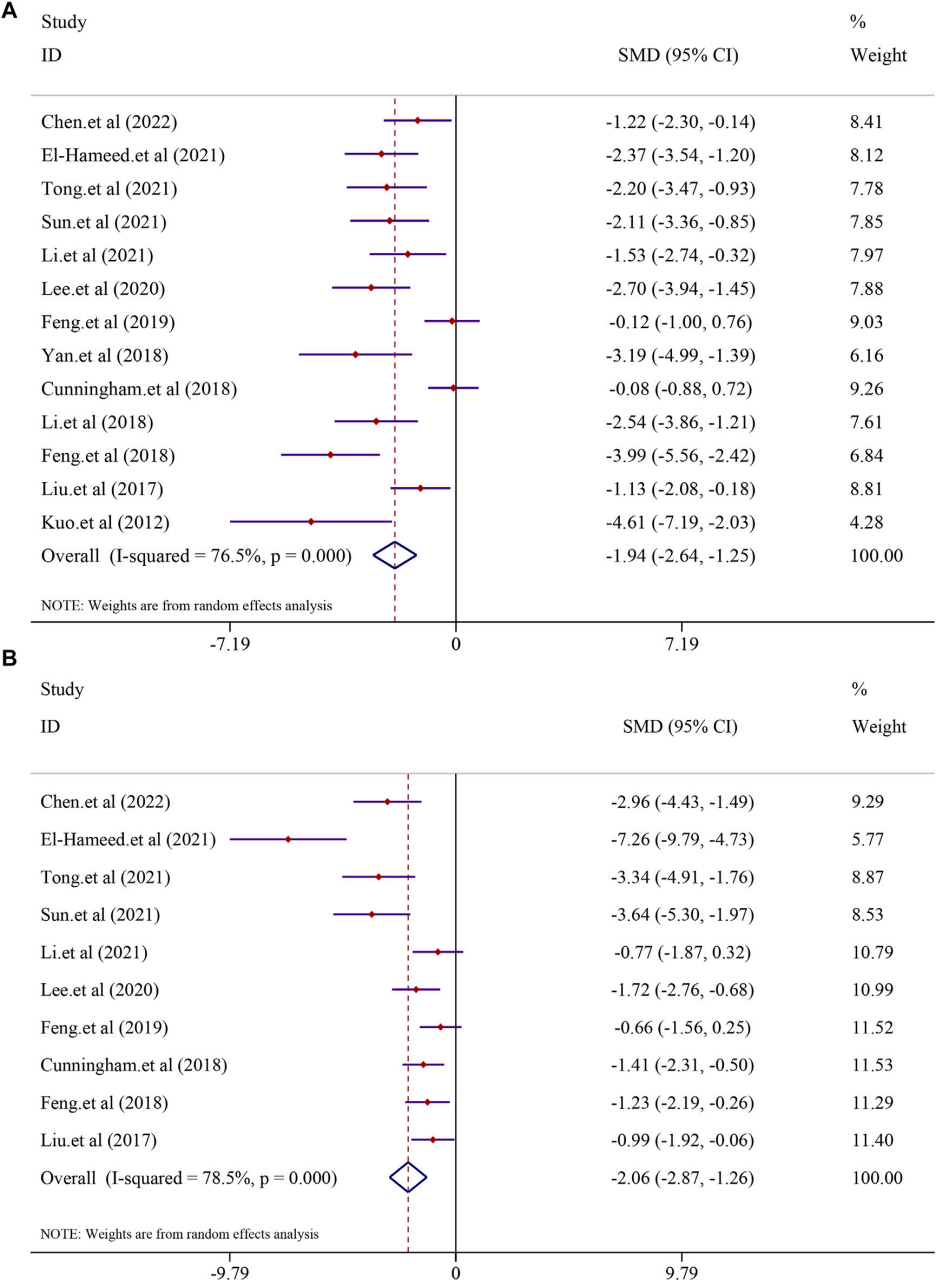
Effect of curcumin on ALT and AST levels in NAFLD studies [**(A)** Pooled effect of ALT; **(B)** Pooled effect of AST].

A total of 13 studies, including 225 animals, reported ALT levels after curcumin treatment (*I* ^2^ = 76.5%; *p* < 0.0001). Significant heterogeneity existed between these studies and was further analyzed using a random-effects model. The results indicated that curcumin was significant in decreasing ALT levels compared to that in the model group [*SMD* = −1.94, 95*% CI* (−2.64, −1.25), *p* < 0.0001] (Figure 3). Analysis of 10 studies involving 186 animals showed the AST levels after curcumin treatment. However, heterogeneity analysis revealed significant heterogeneity (*I* ^2^ = 78.5%; *p* < 0.0001). Thus, the random-effect model was applied to carry out further analysis. The curcumin group demonstrated a remarkable decrease in AST levels [*SMD* = −2.06, 95*% CI* (−2.87, −1.26), *p* < 0.0001] (Figure 3).

#### 3.4.2 The effect on fatty liver indicators

**FIGURE 4:**
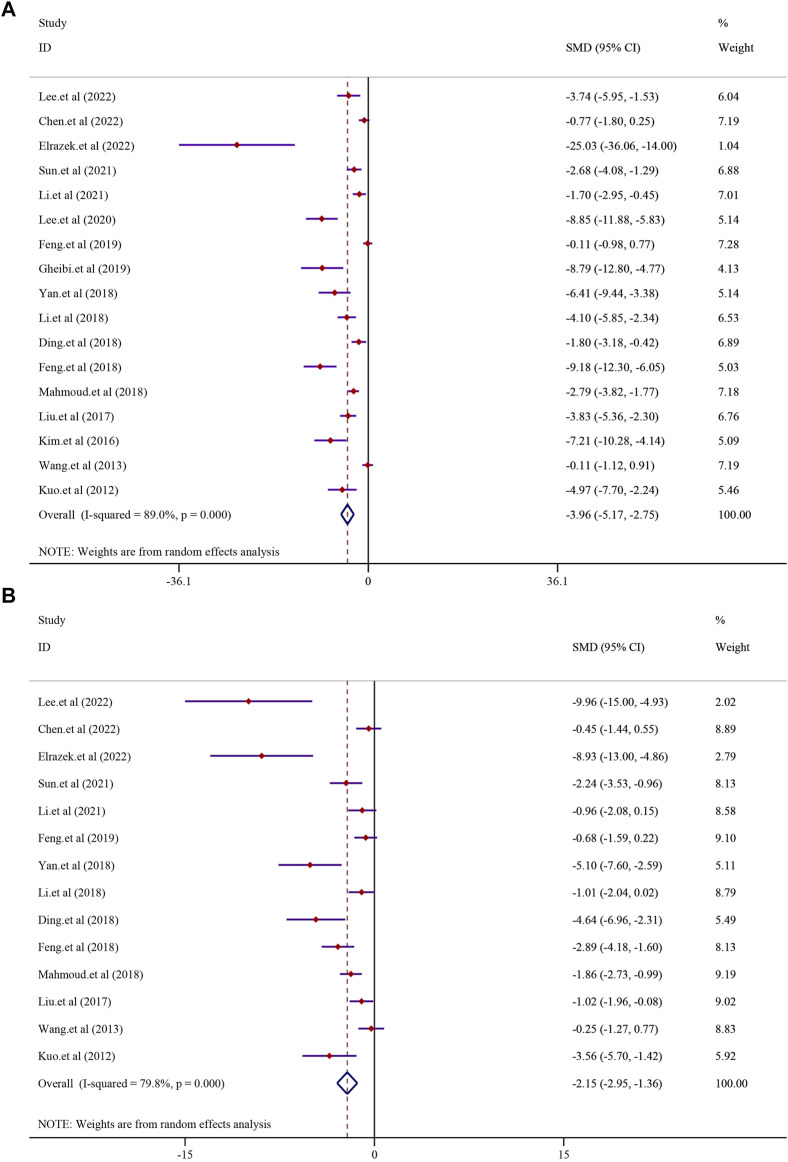
Effect of curcumin on TG and TC levels in NAFLD [**(A)** Pooled effect of TG; **(B)** Pooled effect of TC].

The effect of curcumin on the TG level was reported in 17 trials. Significant heterogeneity existed between these studies and was further analyzed using a random-effects model (*I* ^2^ = 89.0%, *p* < 0.0001); the random-effect model was used to conduct a meta-analysis, and the result demonstrated that there was a significant reduction in the TG level in the curcumin group compared to that in the model group [*SMD* = −3.96, 95*% CI* (−5.17, −2.75), *p* < 0.0001] (Figure 4). The effect of curcumin on the TC levels was evaluated in 14 trials involving 224 animals. Due to the substantial heterogeneity observed, a random-effects model was employed (*I* ^2^ = 79.8%, *p* < 0.0001). The results indicated a significant reduction in TC levels within the curcumin group [SMD = −2.15, 95% CI (−2.95, −1.36), *p* < 0.0001] (see Figure 4). Additionally, 11 studies comprising 170 animals investigated LDL-C levels. Given the significant heterogeneity detected (*I* ^2^ = 79.2%, *p* < 0.0001), a random-effect model was utilized for further meta-analysis. The findings revealed a substantial decrease in LDL-C levels within the curcumin group in comparison to that in the model group [SMD = −1.93, 95% CI (−2.79, −1.08), *p* < 0.0001] (refer to Supplementary Figure S1A). Similarly, 11 studies involving 170 animals were analyzed for HDL-C levels. High heterogeneity among these studies led to the application of a random-effects model (*I* ^2^ = 89.0%; *p* < 0.0001). The meta-analysis suggested a trend of increased HDL-C levels in the curcumin group compared to that in the model group, although it did not reach statistical significance [SMD = 1.21, 95% CI (−0.04, 2.45), *p* = 0.057] (Supplementary Figure S1B).

#### 3.4.3 The effect on serum insulin, glucose, and HOMA-IR

As for the index of serum insulin (*I* ^2^ = 92.4%, *p* < 0.0001), glucose (*I* ^2^ = 89.3%, *p* < 0.0001), and HOMA-IR (*I* ^2^ = 83.2%, *p* = 0.003), curcumin could remarkably reduce the insulin level compared to the model group, but there was no significance [*SMD* = −1.10, 95*% CI* (−3.36, 1.16), *p* = 0.34] (Supplementary Figure S2A). Seven studies with 109 animals demonstrated that treatment with curcumin could significantly reduce the glucose level compared to that in the model group [*SMD* = −3.25, 95% *CI* (−5.09, −1.40), *p* = 0.001] (Supplementary Figure S2B). A similar result was also reflected in HOMA-IR, where three studies containing 49 animals suggested that curcumin could significantly reduce the HOMA-IR index [*SMD* = −3.24, 95*% CI* (−5.55, −0.93), *p* = 0.006] (Supplementary Figure S2C).

### 3.5 Effect of curcumin on liver fibrosis

A meta-analysis of the included literature on curcumin for LF was performed. Indicators of liver function, liver fibrosis indicators, oxidative stress, and inflammation were assessed separately, and the potential tendency of curcumin on these indicators was explored. The results suggest that curcumin may effectively reverse the level of LF through anti-inflammatory and anti-oxidative stress pathways.

#### 3.5.1 The effect on liver function

**FIGURE 5:**
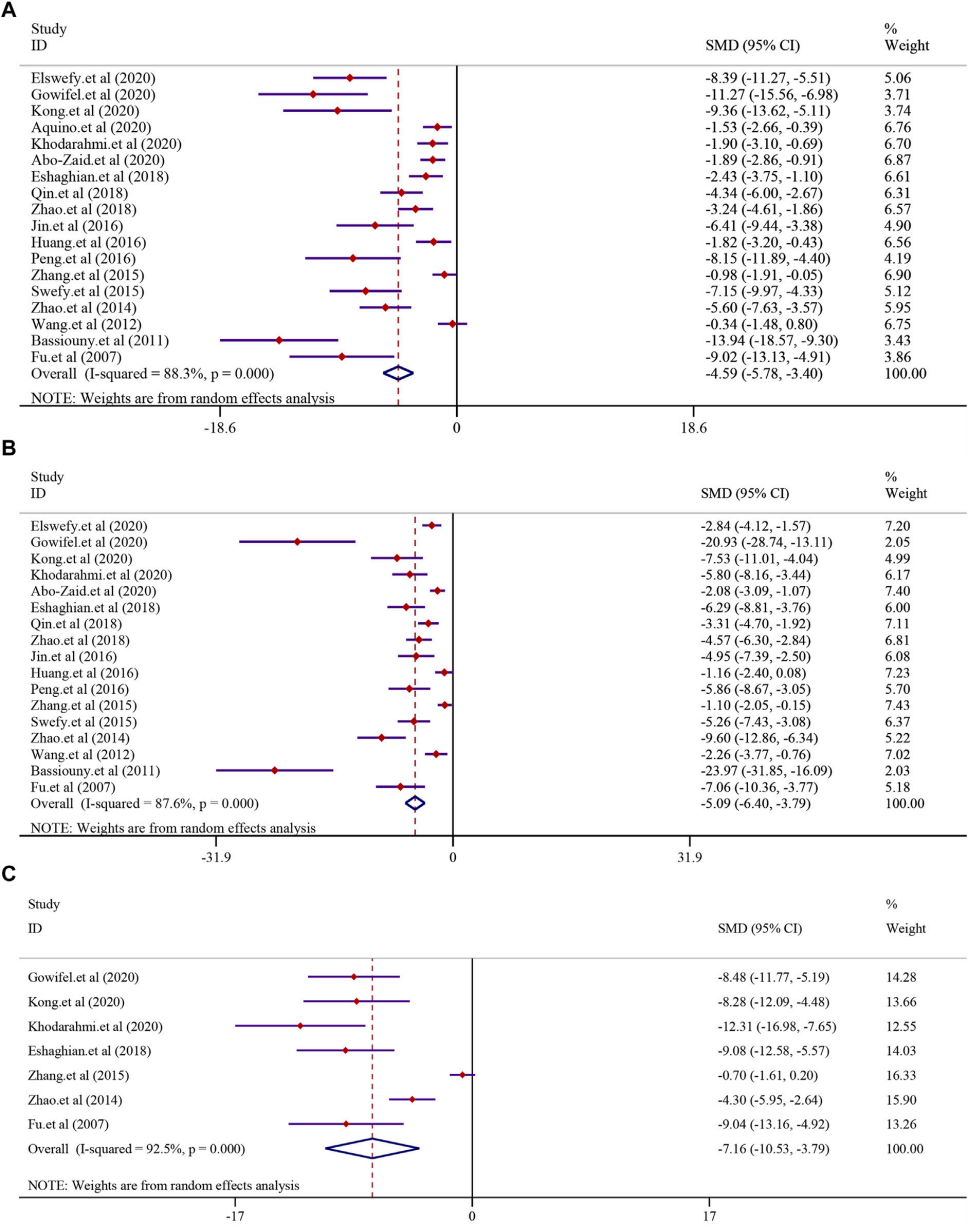
Effect of curcumin on ALT, AST, and ALP levels in liver fibrosis [**(A)** Pooled effect of ALT; **(B)** Pooled effect of AST; **(C)** Pooled effect of ALP].

The effect of curcumin on the ALT level in LF was reported in 18 trials using a random-effect model (*I* ^2^ = 88.3%, *p* < 0.0001). The result indicated that curcumin significantly reduced the level of ALT in LF [n = 296, *SMD* = −4.59, 95% *CI* (−5.78, −3.40), *p* < 0.0001] (Figure 5A). The analysis from 17 studies reported the AST level after treatment with curcumin (*I* ^2^ = 87.6%, *p* < 0.0001). The result showed that curcumin had a significant effect in reducing the level of AST compared to that in the model group [n = 280, *SMD* = −5.09, 95*% CI* (−6.40,-3.79), *p* < 0.0001] (Figure 5B). A similar result was also reflected in ALP, and seven studies were included in this meta-analysis using a random-effect model (*I* ^2^ = 92.5%; *p* < 0.0001). The result showed that curcumin had a significant effect in reducing the level of ALP compared to that in the model group [n = 112, *SMD* = −7.16, 95*% CI* (−10.53, −3.79), *p* < 0.0001] (Figure 5C).

#### 3.5.2 The effect on liver fibrosis

**FIGURE 6:**
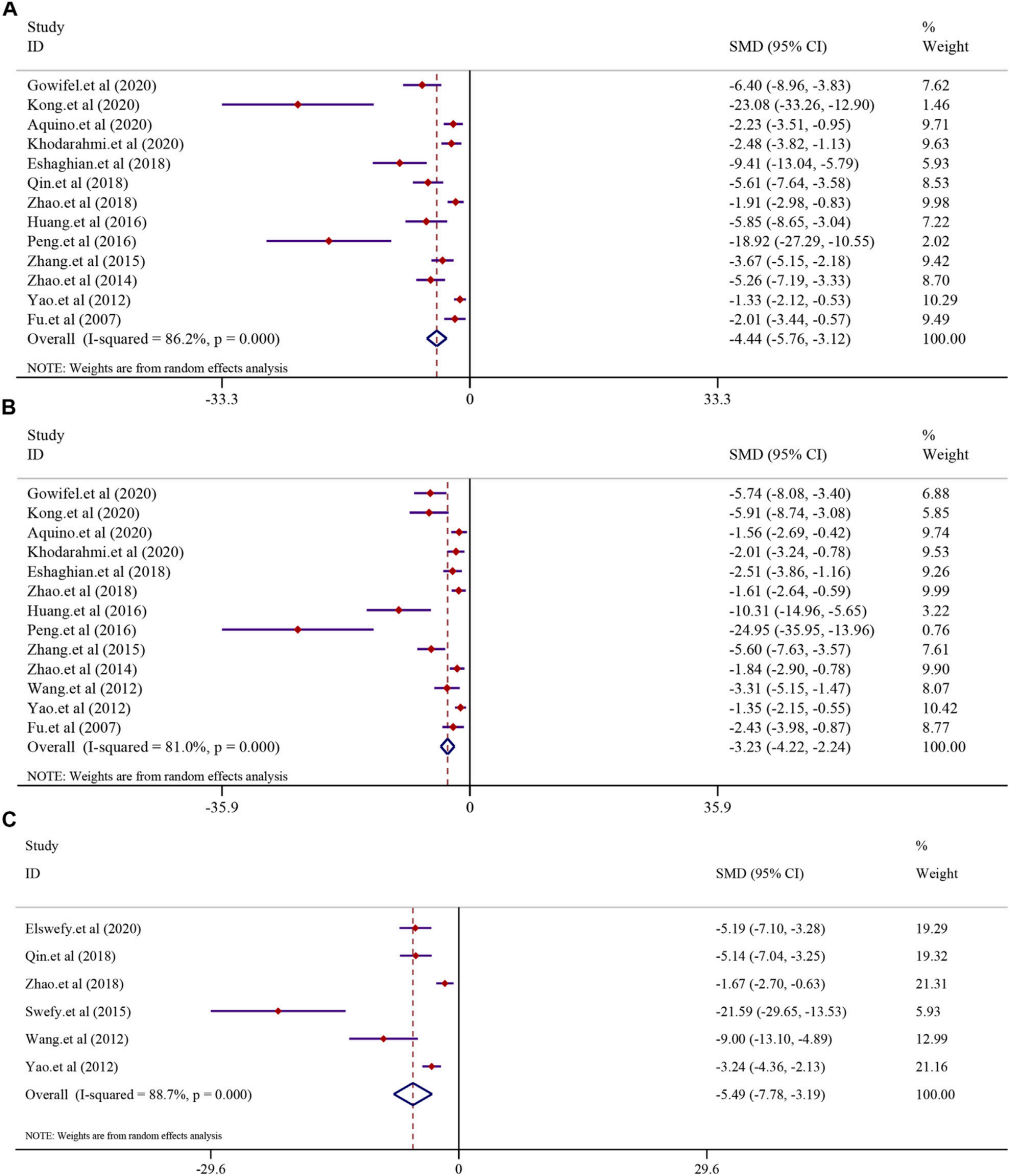
Effect of curcumin on α-SMA, collagen levels, and Area of collagen fibrosis in liver (%) in liver fibrosis [**(A)** Pooled effect of α-SMA; **(B)** Pooled effect of collagen levels; **(C)** Pooled effect of Area of collagen fibrosis in liver (%)].

The effect of curcumin on the α-SMA level, collagen level, area of collagen fibrosis in the liver (%), hydroxyproline level, hyaluronidase (HA) level, the laminin (LN) level, and precollagen type Ⅲ (PCⅢ) levels was tested using a random-effect model. The α-SMA level was reported in 13 trials (*I* ^2^ = 86.2%, *p* < 0.0001). The result showed that curcumin had a significant effect in reducing the level of α-SMA compared to that in the model group [*n* = 222, *SMD* = −4.44, 95*% CI* (−5.76, −3.12), *p* < 0.0001] (Figure 6A). A similar result was also reflected in the collagen level. A total of 13 trials reported the collagen level in this meta-analysis (*I* ^2^ = 81.0%, *p* < 0.0001), and the result showed that curcumin could significantly reduce the collagen level in LF [*n* = 214, *SMD* = −3.23, 95% *CI* (−4.22, −2.24), *p* < 0.0001] (Figure 6B). The analysis from six studies reported the area of collagen fibrosis in the liver (%) after treatment with curcumin (*I* ^2^ = 88.7%, *p* < 0.0001). The result showed that the curcumin group demonstrated a remarkable decrease in the area of collagen fibrosis in the liver (%) [*n* = 118, *SMD* = −5.49, 95*% CI* (−7.78,-3.19), *p* < 0.0001] (Figure 6C). The effect of curcumin on the hydroxyproline level in LF was reported in six trials (*I* ^2^ = 90.9%, *p* < 0.0001). A meta-analysis result revealed that curcumin significantly decreased the hydroxyproline levels in LF [*n* = 84, *SMD* = −8.15, 95% *CI* (−12.17, −4.13), *p* < 0.0001] (Supplementary Figure S3A). The analysis from four studies reported the hyaluronidase (HA) level (*I* ^2^ = 91.0%, *p* < 0.0001). The curcumin group demonstrated a remarkable decrease in the hyaluronidase (HA) level in LF [n = 64, *SMD* = −5.60, 95% *CI* (−9.20, −2.01), *p* < 0.0001] (Supplementary Figure S3B). A total of three studies reported the laminin (LN) level (*I* ^2^ = 91.6%, *p* < 0.0001). The result showed that the curcumin group demonstrated a remarkable decrease in the laminin (LN) levels [*n* = 52, *SMD* = −3.94, 95% *CI* (−7.30, −0.58), *p* = 0.022] (Supplementary Figure S3C). A total of four studies reported PC Ⅲ levels after curcumin treatment. Significant heterogeneity existed between these studies and was further analyzed (*I* ^2^ = 86.3%; *p* < 0.0001). The result indicated that PC Ⅲ was reduced significantly in the curcumin groups compared to the model groups [*n* = 64, *SMD* = −3.20, 95% *CI* (−5.35, −1.04), *p* = 0.004] (Supplementary Figure S3D).

### 3.6 Effect of curcumin on hepatocellular carcinoma

**FIGURE 7:**
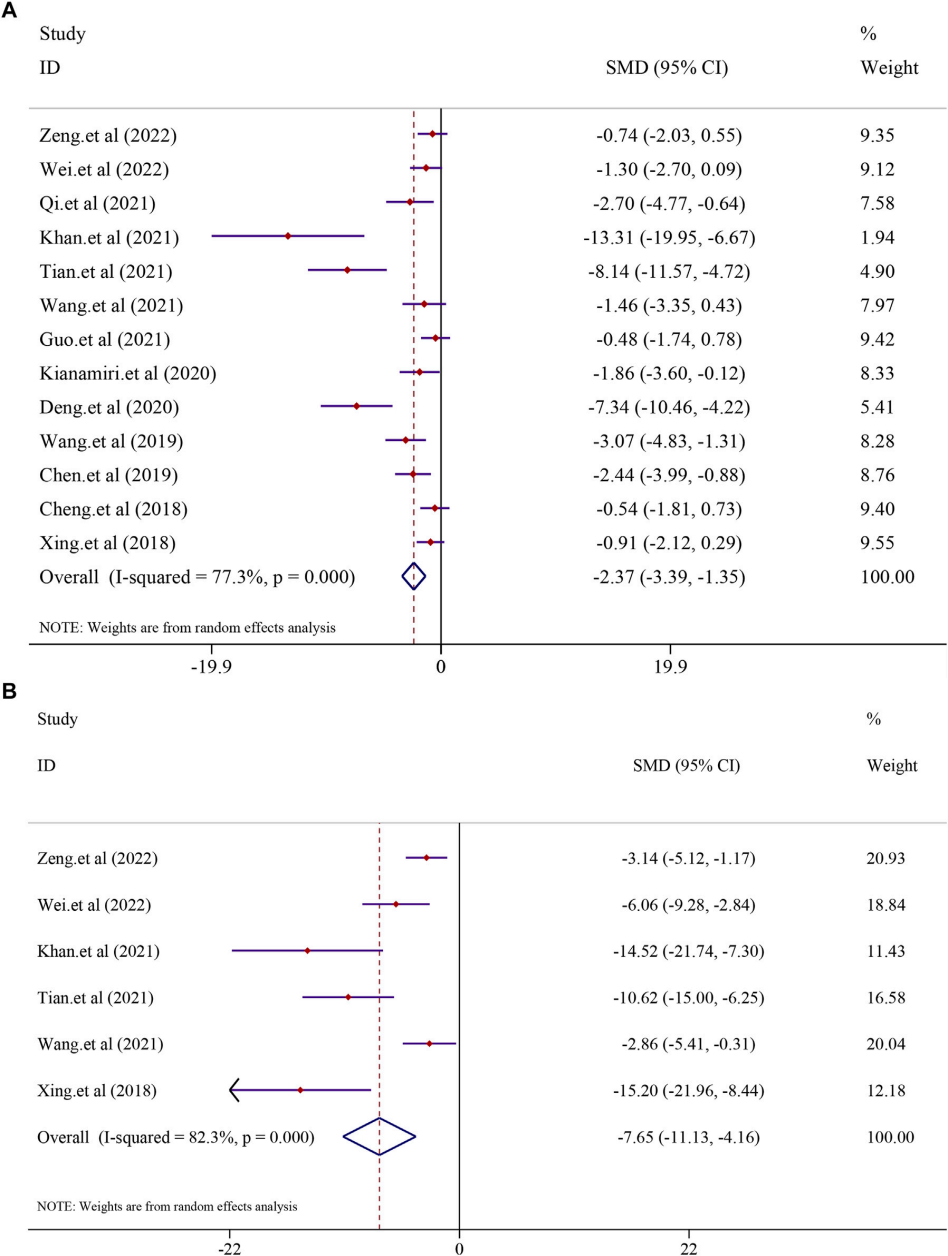
Effect of curcumin on Tumor volume and Tumor weight in liver cancer [**(A)** Pooled effect of Tumor volume; **(B)** Pooled effect of Tumor weight].

All the 13 included studies evaluated tumor volume and weight as the primary criteria of HCC. Thirteen studies on curcumin in the treatment of HCC found that curcumin had a substantial effect on the treatment of hepatocellular carcinoma by lowering the tumor volume and weight. The analysis from 13 studies reported the tumor volume after treatment with curcumin (*I* ^2^ = 77.3%, *p* < 0.0001). The results suggested that curcumin had a significant inhibitory effect on tumor volume compared to that in the model group [*n* = 136, *SMD* = −2.37, 95*% CI* (−3.39,-1.35), *p* < 0.0001] (Figure 7A). A similar result was also reflected in tumor weight, and the effect of curcumin on tumor weight was reported in six trials using a random-effect model (*I* ^2^ = 82.3%, *p* < 0.0001). The results revealed that curcumin had a significant inhibitory effect on reducing the tumor weight compared to that in the model group [*n* = 62, *SMD* = −7.65, 95*% CI* (−11.13,-4.16), *p* < 0.0001] (Figure 7B).

### 3.7 The consistent regulatory role of curcumin in the liver diseases

In the process of disease progression, there are a few indicators that can span the entire course and accurately reflect the disease’s advancement. However, cytokines such as TNF-α, IL-6, IL-1β, MDA, and GSH interact during the progression from NAFLD to LF and then to HCC, forming a complex network of inflammation and immune regulation. Their overexpression and interactions drive the progression of the disease, simultaneously providing valuable targets for the treatment of NAFLD, LF, and liver cancer. Therefore, studying and intervening in the activities of these cytokines are of paramount importance for exploring disease mechanisms and developing novel therapeutic approaches. Therefore, we analyzed the key indicators for these three stages of diseases and found that curcumin can improve various outcome measures. These results indicate that curcumin exhibits promising therapeutic effects across the spectrum of NAFLD, LF, and liver cancer. Upon analysis, we observed that curcumin consistently regulates TNF-α, IL-6, IL-1β, MDA, and GSH, regardless of the disease state. Subsequent analysis of common downstream factors in these three diseases revealed that curcumin primarily exerts its therapeutic effects on a range of liver conditions by modulating inflammatory factors such as TNF-α, IL-6, and IL-1β, as well as oxidative stress factors MDA and GSH. This suggests that curcumin holds the potential to be a comprehensive hepatoprotective agent.

#### 3.7.1 Inflammatory indexes

**FIGURE 8:**
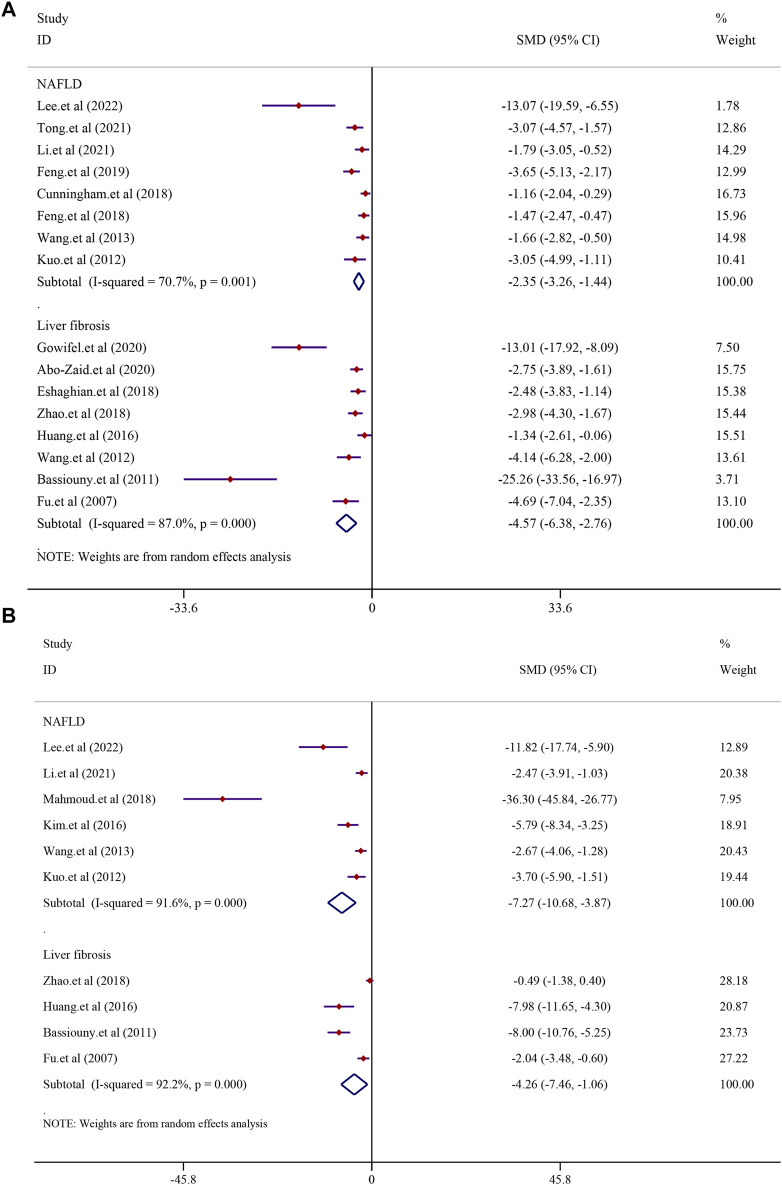
Effect of curcumin on TNF-α and IL-6 levels in NAFLD and Liver fibrosis [**(A)** Pooled effect of TNF-α; **(B)** Pooled effect of IL-6].

There were eight studies that reported the TNF-α level in NAFLD models. The results demonstrated that treatment with curcumin could significantly reduce the TNF-α level compared to that in the NAFLD model group [*SMD* = −2.35, 95% *CI* (−3.26, −1.44), *p* < 0.0001]. In addition, eight studies reported the TNF-α level in liver fibrosis models, and the results demonstrated that treatment with curcumin could significantly reduce the TNF-α level compared to that in the liver fibrosis model group [*n* = 132, *SMD* = −4.57, 95% *CI* (−6.38, −2.76), *p* < 0.0001] (Figure 8A). The IL-6 levels were found to be significantly lower in the curcumin-treated groups than in the model groups. Curcumin was effective in both the NAFLD model [*SMD* = −7.27, 95% *CI* (−10.68, −3.87), *p* < 0.0001] and the LF model [*n* = 64, *SMD* = −4.26, 95% *CI* (−7.46, −1.06), *p* = 0.009] (Figure 8B). Curcumin significantly reduced the levels of IL-1β in NAFLD models [*SMD* = −2.60, 95% *CI* (−3.31, −1.90), *p* < 0.0001] and LF models [n = 52, *SMD* = −2.72, 95% *CI* (−3.93, −1.51), *p* < 0.0001] (Supplementary Figure S4).

#### 3.7.2 Oxidative stress indexes

**FIGURE 9:**
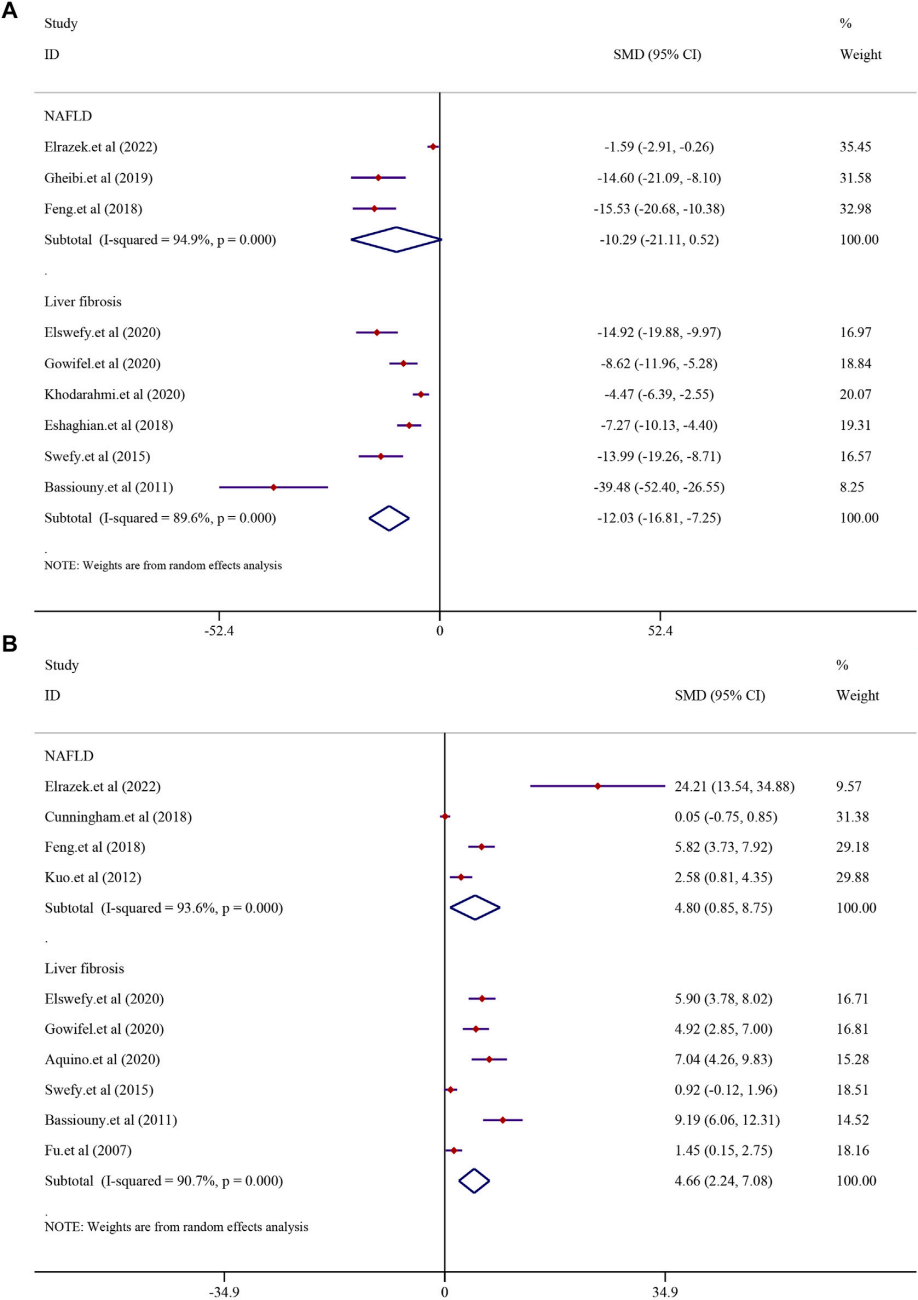
Effect of curcumin on MDA and GSH levels in NAFLD and Liver fibrosis [**(A)** Pooled effect of MDA; **(B)** Pooled effect of GSH].

In order to investigate the effect of curcumin on antioxidation in NAFLD and LF models, MDA was analyzed. Three studies reported MDA in NAFLD models, and six studies reported it in LF models. The results demonstrated that treatment with curcumin could significantly reduce the MDA level compared with treatment in the NAFLD model group [*SMD* = −10.29, 95% *CI* (−21.11, 0.52), *p* = 0.062]. A similar result was also reflected in LF models [*n* = 104, *SMD* = −12.03, 95% *CI* (−16.81, −7.25), *p* < 0.0001] (Figure 9A). GSH levels were found to be significantly higher in the curcumin-treated groups than in the model groups. Curcumin was effective in both the NAFLD model [*SMD* = 4.80, 95% *CI* (0.85, 8.75), *p* = 0.017] and LF model [*n* = 100, *SMD* = 4.66, 95% *CI* (2.24, 7.08), *p* < 0.0001] (Figure 9B).

#### 3.7.3 Curcumin inhibits cancer occurrence by suppressing inflammation-induced carcinogenesis and oxidative stress

In the process of inflammation-induced carcinogenesis, inflammation stands out as the crucial factor driving this progression. Curcumin plays a significant role in inhibiting this process by suppressing the inflammatory pathways, reducing the production of inflammatory factors, and alleviating tissue inflammation reactions, thus hindering the onset of inflammation-induced cancer. Moreover, curcumin also has the capability to modulate cellular signaling pathways, intervening in the proliferation and survival of inflamed cancer cells. It might achieve this by influencing cell-cycle regulation and apoptosis signaling pathways, or inhibiting the expression of tumor-related genes, ultimately restraining the abnormal proliferation of inflamed cancer cells. Additionally, curcumin is believed to exert a certain influence on tumor stem cells, which play a pivotal role in the development of inflammation-induced cancer. Curcumin possibly achieves this by regulating the characteristics of stem cells, thereby inhibiting the formation of inflammation-induced cancer. In the NAFLD stage, oxidative stress factors such as GSH and MDA are commonly abnormal in NAFLD patients, leading to cellular lipid peroxidation and exacerbating liver cell damage. In the stages of LF and HCC, persistent oxidative stress injury may cause DNA damage and cell apoptosis, driving the progression of LF, and curcumin may inhibit the development of this process.

### 3.8 The dosage and duration range analysis in preclinical studies

**FIGURE 10:**
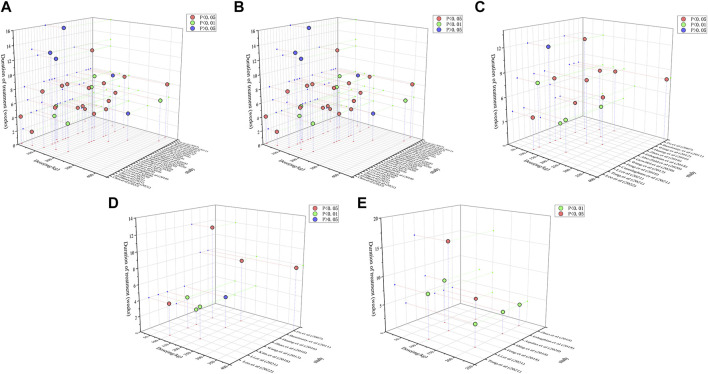
3D dose/time-effect images. **(A)** ALT; **(B)** AST; **(C)** TNF-α; **(D)** IL-6; **(E)** IL-1β.

Dose–response relationships and time-effects play an influential role in clinical pharmacotherapy. To investigate whether the dose and duration of curcumin administration affect the therapeutic intervention, we generated three-dimensional (3D) images (Figure 10) based on the experimental dose and duration effects to determine the optimal duration and the most appropriate dosage of curcumin in the three different disease stages. For hepatoprotective effects, a clinicopathological syndrome characterized by AST and ALT levels, we selected AST and ALT as the main indicators. For the common indicators across the three diseases, we selected TNF-α, IL-6, and IL-1β levels as the primary markers to reflect the inhibitory effects of curcumin on inflammation-induced carcinogenesis. Moreover, oxidative stress markers MDA and GSH are integral throughout the progression of these diseases. Therefore, we have also investigated the dose–response relationship of curcumin’s effects on MDA and GSH (Table 2).

**TABLE 2:** Optimal duration and dosage of administration for the key factors.

Indicator	Disease	Duration (weeks)	Dosage (mg/kg)
ALT	NAFLD	2–8	50–200
	LF	4–8	100–400
AST	NAFLD	4–8	50–200
	LF	4–8	100–400
TNF-α	NAFLD	4–12	100–200
	LF	4–12	100–400
IL-6	NAFLD	2–4	100–200
	LF	4–12	100–400
IL-1β	NAFLD	4–8	60–200
	LF	4–16	100–200
GSH	NAFLD	4–8	100–200
	LF	4–8	100–400
MDA	NAFLD	4–8	100–200
	LF	2–8	100–200

#### 3.8.1 Dose–time-effect relationship of curcumin on hepatoprotective indexes

We performed a dose–duration-effect analysis based on the administration dose and time effects to determine the optimal duration and most appropriate dose of curcumin, and doses of 50 mg/kg/d to 200 mg/kg/d of curcumin significantly reduced ALT levels (Figure 10A). However, one study reported that curcumin was still effective at doses less than 10 mg/kg/d, and additional trials are needed to determine this. With respect to treatment duration, 3D images showed curcumin to be ineffective at more than 12 weeks of intervention, while the ALT levels were significantly better at 2 weeks–8 weeks of intervention. As for the AST levels, curcumin at doses of 50 mg/kg/d to 300 mg/kg/d significantly reduced the AST levels (Figure 10B). Similar to ALT, 3D images showed that curcumin was ineffective at more than 12 weeks, while the AST levels improved significantly at 4–8 weeks of intervention. Therefore, our findings suggest that curcumin interventions of 4 to 8 weeks at doses of 50–200 mg/kg may be the best for hepatoprotective indexes ALT and AST.

#### 3.8.2 Dose–time-effect relationship of curcumin on inflammatory factors

We selected TNF-α, IL-6, and IL-1β levels as the main indicators of curcumin’s inhibition of inflammatory cancer transformation, which could reflect hepatoprotective effects. Curcumin can significantly decrease TNF-α levels at doses of 100–400 mg/kg for 4–12 weeks (Figure 10C). Similarly, the IL-6 levels can be significantly reduced by curcumin at the dosage of 100–400 mg/kg and the duration of 4–12 weeks. As for IL-1β levels, curcumin was effective at doses of 60–200 mg/kg for 4–16 weeks of intervention (Figures 10D, E). In general, curcumin effectively alleviated inflammatory cancer transformation at a dosage of 100–400 mg/kg and a duration of 4–12 weeks.

#### 3.8.3 Dose–time-effect relationship of curcumin on oxidative stress factors

The oxidative stress markers, serving as the primary indicators of liver damage, can reflect the extent of progression in liver diseases. The dose–duration-effect analysis result showed that the optimal administration period for curcumin is 4–8 weeks, with the ideal dosage range being 100–400 mg/kg. Under these conditions, it effectively elevates the levels of GSH. Additionally, the optimal administration period for alleviating MDA levels falls within 2–8 weeks, with the best dosage concentrated between 100 and 200 mg/kg (Supplementary Figure S5).

### 3.9 Sensitivity test and publication bias

To further explore the stability of the results, sensitivity analyses were conducted. After removing the literature one by one, the results revealed that the effects of curcumin on liver diseases were stable. The details of all sensitivity analyses are shown in Supplementary Figure S6. Publication bias analysis was performed for the main indicators, including ALT, AST, TNF-α, IL-6, and IL-1β (Supplementary Figure S6). However, the results showed potential publication biases existed regarding the effects of curcumin on ALT (Egger’s test, *p* < 0.001), AST (Egger’s test, *p* < 0.001), TNF-α (Egger’s test, *p* < 0.001), IL-6 (Egger’s test, *p* < 0.001), and IL-1β (Egger’s test, *p* < 0.005) (Supplementary Figure S7).

### 3.10 Summary of the mechanism of curcumin in the treatment of liver diseases

The mechanisms of curcumin in the inhibition of the transition from NAFLD to HCC are multiple, well-established, and multi-targeted. The phenotypes involved are mainly related to oxidative stress, inflammation, and apoptosis (Supplementary Table S2).

#### 3.10.1 Summary of the mechanism of curcumin in the treatment of NAFLD

The possible molecular mechanism of curcumin on NAFLD was also summarized according to preclinical studies. It is characterized by network regulation with multiple bioactive targets. Many studies have proved that curcumin could activate the AMPK/Sirt1 signaling pathway. Furthermore, curcumin activates the AMPK/SIRT1 signaling pathway and regulates ACC-CPT-1 and SREBP1-FASN to alleviate hepatic steatosis by suppressing 11 beta-hydroxysteroid dehydrogenase (11β-HSD1). In addition, curcumin regulates nuclear factor erythroid 2-related factor 2 (Nrf2) changes in gene expression to reduce adipogenesis, endoplasmic reticulum (ER) stress, inflammation, oxidative stress, and fibrosis. This pathway was crucial during NAFLD progress. Curcumin ameliorates steatosis by phosphorylating adenosine 5′-monophosphate (AMP)-activated protein kinase (AMPK) and thereby inhibiting preadipocyte differentiation, inhibiting downstream target proteins CCAAT-enhancer-binding proteins (C/EBP), peroxisome proliferator-activated receptor γ (PPARγ), and sterol regulatory element-binding protein-1c (SREBP-1c) and decreasing hepatic TG levels. Meanwhile, curcumin blocks hepatic adipogenesis and TG accumulation by regulating lipogenic and autophagy-related genes and inhibiting apoptosis, thereby improving NAFLD and insulin resistance. Second, curcumin demonstrated the ability to reverse inflammation-related signals. It achieved this by suppressing toll-like receptor 2 (TLR-2) and toll-like receptor 4 (TLR-4), thereby inhibiting nuclear factor kappa-B (NF-κB) and mitogen-activated protein kinase (MAPK) signal pathways, leading to a reduction in inflammatory cytokines. Additionally, curcumin played a role in preventing HFD-induced hepatic steatosis by enhancing the intestinal barrier function and modulating the TLR4/NF-κB hepatic inflammatory pathways. The inhibition of macrophage activation, specifically M1 macrophages, by curcumin further resulted in a reduction of IL-1β and TNF-α expression, contributing to the alleviation of liver dysfunction and inflammation during NAFLD.

#### 3.10.2 Summary of the mechanism of curcumin in the treatment of liver fibrosis

Curcumin inhibits oxidative stress by activating peroxisome proliferator-activated receptor alpha (PPAR-α) to inhibit the release of reactive oxygen species (ROS) from hepatocytes, increases hepatic glutathione levels within normal values to reduce oxidative stress, leads to a decline in the lipid hydroperoxide content, and regulates autophagy through the AMPK and PI3K/AKT/mTOR signaling pathways, leading to elevated autophagic blood flow in hepatocytes. Curcumin also effectively reduced EMT in hepatocytes by blocking the TTC3/SMURF2/SMADs axis and inhibited extracellular matrix (ECM) production by activating autophagy. In addition, curcumin significantly upregulated Nrf2/HO-1 gene expression, downregulated NF-κB and TGF-β, and phosphorylated smad3 protein expression as well as α-SMA and collagen-1 gene expression, ameliorating inflammation and oxidative stress by regulating the Nrf2/HO-1, NF-κB, and TGF-β/Smad3 signaling pathways. Furthermore, curcumin exerts antifibrotic effects by inhibiting hepatic stellate cell activation and reducing the expression levels of α-SMA and SMAD family member 3 (Smad3). The normalization of the TGF-β -Smad3 pathway leads to an upregulation of the Smad7 levels, inhibiting the production of NF-κB-related pro-inflammatory cytokines and reducing the presence of activated hematopoietic stem cells in the liver parenchyma. Curcumin facilitates apoptosis, decreasing the number of necrotic cells during chronic liver injury by upregulating Bax expression and downregulating Bcl-2 mRNA, thereby mitigating the inflammatory response associated with cell apoptosis. Moreover, it hinders the proliferation of DNA-damaged hepatocytes, preventing their progression to HCC.

#### 3.10.3 Summary of the mechanism of curcumin in the treatment of hepatocellular carcinoma

Curcumin exerts its anticancer effects through three main mechanisms: 1. regulating the expression of apoptosis-related proteins; 2. inhibiting the cell cycle process; 3. inhibiting the NF-κB pathway. Apoptosis is regarded as one of the most powerful strategies to combat the growth of cancer cells. Many *in vitro* and *in vivo* studies have reported that curcumin promotes apoptosis as a consequence of regulating the expression of apoptosis-related proteins. Curcumin can regulate multiple signaling targets in cancer cells, leading to upregulation of the pro-apoptotic gene Bax and caspase cascade response and downregulation of the anti-apoptotic gene Bcl-2 (Mortezaee et al., 2019). For example, curcumin-treated HepG2 cells can promote apoptosis through suppressing Bcl-2 and upregulating Bax protein expression (Qu et al., 2020). In addition to the participation of curcumin in apoptosis, it can also arrest the cell cycle process. Many studies have reported curcumin-mediated cell cycle arrest as a consequence of regulation by cell cycle regulators, including the upregulation of cell cycle protein-dependent kinase inhibitors (CDKIs) and the downregulation of cell cycle protein B1, cell cycle protein D, cell cycle protein E, and Cdc2. Curcumin regulates the downregulation of these proteins, thereby causing cell cycle arrest in the G1/S and G2/M phases (Lee DS. et al., 2009). The reduction in the level of cyclin D1 leads to cell cycle block in the G1/S phase. cdc2/cell cyclin B complex regulates the progression of the g2 phase to M phase, while curcumin treatment decreases cell cyclin D1 and CDC2/cell cyclin B complex. This leads to the conclusion that curcumin causes cancer cell death through cell cycle arrest in the G1/S and G2/M phases (Liczbiń et al., 2020). Inflammation is also an essential contributor to the development of HCC, and NF-κB is closely associated with inflammation and cancer progression as a transcription factor. Curcumin has been reported to exert anti-tumor effects by acting as a validated NF-κB target inhibitor. NF-κB-induced tumor invasion and metastasis are mediated by matrix metalloprotein 9 (MMP9) as NF-κB contains MMP9-binding sites. NF-κB activation can upregulate MMP-2 and MMP-9 and, thus, promote HCC metastasis (Jung et al., 2019). Curcumin has been reported to inhibit NF-κB expression and the activity of MMP-2 and MMP9, thereby suppressing tumor metastasis and invasion (Deng et al., 2020).

## 4 Discussion

### 4.1 Curcumin for liver diseases

**FIGURE 11:**
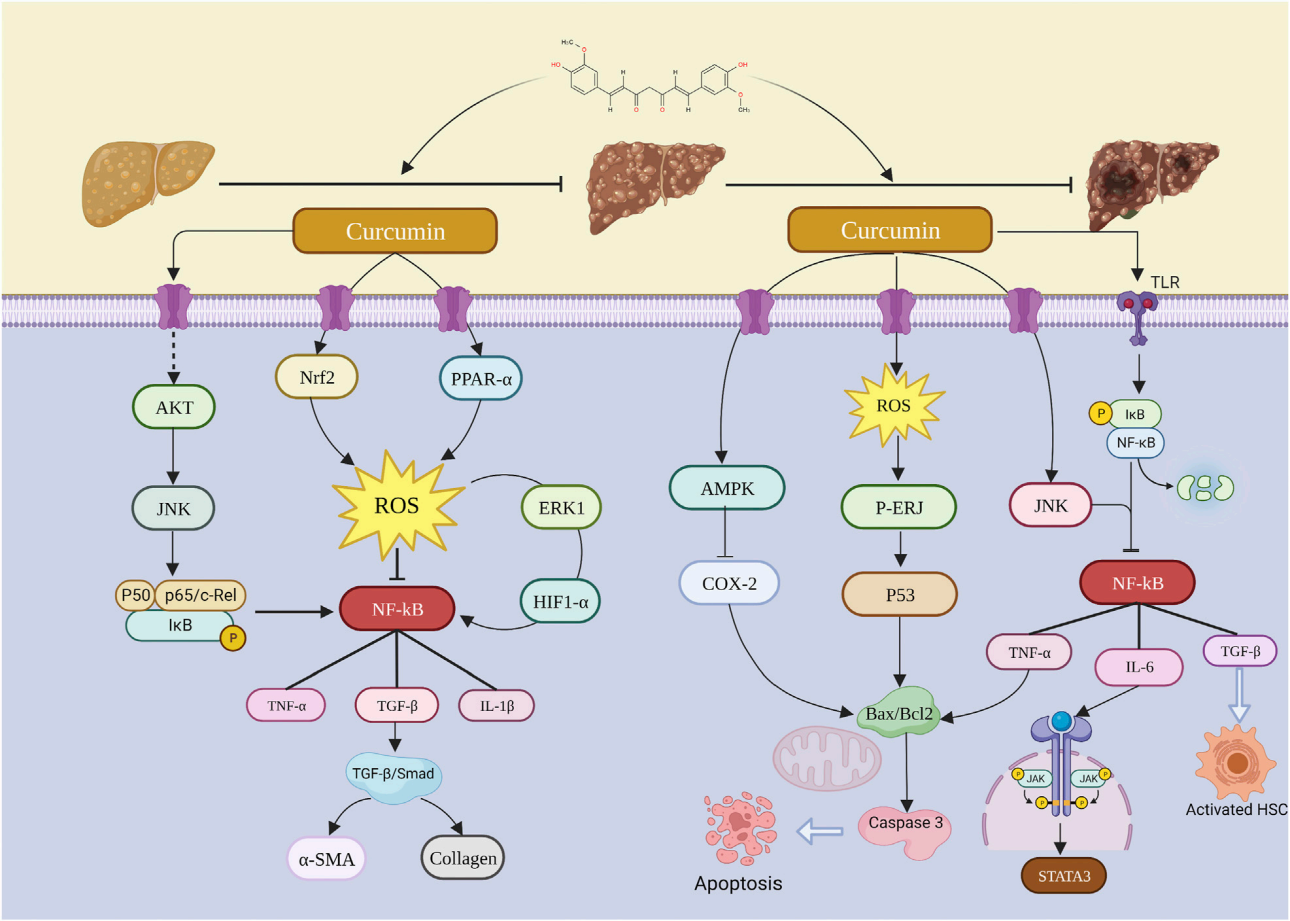
Possible molecular mechanism of curcumin on the progression of NAFLD–LF–HCC.

Regarding NAFLD, we specified TG and TC as the primary indicators of fatty liver and AST, ALT, LDL, HDL, MDA, GSH, TNF-α, IL-6, and IL-1β as the secondary outcome indicators for quantitative analysis and found that curcumin intervention at doses of 50–200 mg/kg for 4 to 16 weeks significantly reduced TG and TC levels and significantly improved NAFLD-induced oxidative stress and inflammatory factors. As for LF, we designated α-SMA, collagen level, and hydroxyproline as the primary indicators of fibrosis, and AST, ALT, ALP, hyaluronidase (HA), laminin (LN), TGF-β, GSH, MDA, TNF-α, IL-6, and IL-1β were quantified as secondary outcome indicators. The results indicated that curcumin at 100–400 mg/kg doses for 4–8 weeks significantly reduced the levels of α-SMA, collagen level, and hydroxyproline and significantly ameliorated the oxidative stress-induced elevation of MDA and reduction of GSH, as well as the inflammation-induced elevated expression of TNF-α, IL-6, and IL-1β. In addition, according to the hepatocellular carcinoma literature we included, curcumin suppresses the development of transplanted tumors in animals, which significantly suppressed the tumor volume and weight compared with that in the model group. Based on quantitative analysis results, we found that curcumin improved liver function indicators, oxidative stress factors, and inflammatory factors in both NAFLD and LF. Therefore, anti-oxidative stress and anti-inflammation may be important ways to block the transition from NAFLD to LF. In addition, the regulation of apoptosis is also an overwhelming contributor to liver disease progression (Figure 11).

### 4.2 Potential synergistic mechanism of curcumin on the transition of NAFLD–LF–HCC

#### 4.2.1 Inflammation as a promoter of liver disease

..Major pro-inflammatory mechanisms underlie the transition from NAFLD to LF. The main cause of NAFLD is the expansion of fat depots and the accumulation of ectopic fat due to nutrient overload. In the presence of macrophage infiltration, a pro-inflammatory state develops in visceral adipose tissue. Activation of TLR4 receptors in Kupffer cells and hepatocytes further activates and phosphorylates NF-κB, which induces cytokine and chemokine production by NOD-like receptor thermal protein domain-associated protein 3 (NLRP3) inflammatory vesicles (Mazo et al., 2013). These cytokines/chemokines accelerate the progression of non-alcoholic fatty liver to NASH by binding to specific receptors in the hepatocytes and immune response cells and then promoting inflammation and hepatocyte apoptosis. With increased adipogenesis impairing its metabolic capacity, imbalances in lipid metabolism lead to oxidative stress, inflammatory vesicle transcriptional activation, and damaged cell death, which, in turn, stimulates inflammation, tissue regeneration, and fibrosis (Sanyal, 2019). Overproduction of inflammatory cytokines such as TNF-α, interleukin IL-1, and IL-6 causes inflammatory infiltration in the liver, and these induce accelerated progression of NAFLD to NASH (Kumar and Mahato, 2015). In addition, a sustained increase in free fatty acids (FFA) in the hepatocytes leads to increased production of ROS, thereby initiating oxidative stress, which further induces Kupffer cells to produce various cytokines that promote cellular inflammation and apoptosis. These cellular chemokines, specifically TGF-β, promote liver fibrosis by activating hepatic stellate cells (HSC). In contrast, activation of hepatic stellate cells produces substantial amounts of collagen, which promotes excessive deposition of ECM in the liver, leading to the development and progression of LF. The TLR4/NF-κB inflammatory signaling pathway is involved in inducing the expression of multiple inflammatory cytokines in cancer (Rafa et al., 2017). Inflammation promotes the recruitment and activation of myeloid-derived suppressor cells (MDSCs), and activated MDSCs play a critical role in tumorigenesis, progression, and metastasis (Cheng et al., 2020). Inflammation leads to the interaction of activated TLR4 with MyD88, which then induces the phosphorylation of IKK, further promoting the activation of NF-κB, which, in turn, promotes the expression of related genes in the inflammatory signaling pathway (Yao et al., 2019). The TLR4 axis can regulate tumor growth and metastasis by enhancing the immunosuppressive function of MDSCs (Tachibana, 2018). Therefore, curcumin inhibits the expression of MDSCs through the TLR4/NF-κB signaling pathway, which inhibits the growth of HCC and, thus, the vascular endothelial growth factor (VEGF) (Tian et al., 2021). In addition, NF-κB induces the release of the inflammatory factor IL-6, leading to the signal transducer and activator of STAT3 activation, which promotes hepatocyte repair and replication capacity and inhibits apoptosis, thereby promoting the development of hepatocellular carcinoma (Ringelhan et al., 2018). Patients with HCC also normally have elevated IL-6 levels and excessive activation of the signal transducer and activator of transcription 3 (STAT3) (Aleksandrova et al., 2014). However, the administration of curcumin effectively inhibited the IL-6 levels and STAT3 activation, thereby suppressing hepatocellular carcinogenesis (Y et al., 2019).

#### 4.2.2 Oxidative stress is the common basis of various liver diseases

Oxidative stress caused by increased production of ROS is known to play a major role in the pathogenesis of NASH. Hepatocytes are defended against oxidative stress-induced damage through a series of antioxidant pathways. The development of an imbalance between these pro-oxidants and antioxidants leads to a sustained activation of oxidative stress, which becomes a major contributor to the further development of NAFLD into LF. Hepatocytes release excess ROS, which activate Kupffer cells to produce fibrogenic cytokines, such as TNF-α and TGF-β (Trautwein et al., 2015). In addition to this, neutrophils and monocytes can also produce ROS, which stimulate the proliferation of hepatic stellate cells and collagen synthesis, which then leads to LF. Curcumin improves NAFLD through oxidative stress and increases hepatic free GSH levels and improves free radical scavenging through upregulation of the NRF2 gene. This blocks the further development of NAFLD into LF (Cunningham et al., 2018). Some studies have shown that curcumin activates PPAR-α to reduce ROS levels and oxidative stress, increases GSH levels in hepatocytes, and inhibits the TGF-β1/Smad pathway to improve LF (Kong et al., 2020). In addition, oxidative stress leads to activation of the NF-κB pathway through the activation of hepatic stellate cells, which, in turn, promotes the progression of LF to HCC (Urtasun et al., 2008). Curcumin can ameliorate oxidative stress by inhibiting the activity of CAT and SOD and reduce the expression of transforming growth factor TGF-β and tumor necrosis factor TNF-α by inhibiting the activation of NF-κB, thus improving LF and preventing the development of HCC (Hu et al., 2023).

#### 4.2.3 Apoptosis is a double-edged sword

Curcumin may improve HCC by promoting apoptosis, but interestingly, in the treatment of NAFLD and LF, curcumin is used to prevent the progression of NAFLD to LF by inhibiting apoptosis. SREBP1 is a transcription factor for hepatic fatty acid synthases, and they induce steatosis by increasing hepatic TG accumulation, while PPAR-γ leads to increased lipid synthesis by promoting SREBP1 overexpression. Curcumin can exert anti-lipid synthesis effects by suppressing the PPAR-γ and SREBP1 expression (Lee et al., 2020). Meanwhile, Bcl2 and Bax proteins are the main regulators of apoptosis, regulating caspase-induced apoptosis and promoting cell survival. It has been shown that NAFLD leads to overexpression of p53 and caspase 3, as well as reduction of Bcl-2 expression/synthesis. Curcumin administration significantly decreased the p53 and caspase 3 expression, decreased Bax, and upregulated Bcl2 protein expression to inhibit apoptosis and promote hepatocyte survival (Gheibi et al., 2019). In addition, curcumin ameliorates NAFLD-induced apoptosis and steatosis by upregulating GSH-Px and SOD to improve oxidative stress and increase TAC levels (Derdak et al., 2013). LF induces apoptosis in hepatocytes by disturbing the balance between pro-apoptotic and anti-apoptotic proteins. Oxidative stress is also an important factor in apoptosis. It has been suggested that apoptosis of hepatocytes may directly accelerate fibrogenesis (Guicciardi and Gores, 2005). Studies have shown that curcumin exerts its anti-apoptotic effect on LF by decreasing caspase-3 levels and increasing Bcl-2 expression in the liver. TGF-β is a caspase-3 mediated apoptosis inducer, and Awad and El-Sharif demonstrated that curcumin exerts its anti-apoptotic effect through the inhibition of TGF-β in kidney and lung tissues (Awad and El-Sharif, 2011). In addition, during hepatic oxidative stress, curcumin inhibits the mitochondrial translocation and subsequent intrinsic mitochondrial apoptotic pathway of Bax, thereby preventing Bax-mediated Bcl2 inactivation and inhibiting the development of hepatic fibrosis (Elswefy et al., 2020). Since the p53 tumor suppressor plays one of the most crucial roles in triggering apoptosis in cancer cells, the activation of p53 is considered an important mechanism for tumor amelioration. It has been shown that curcumin activation of p53 leads to Bax upregulation and mitochondrial damage, resulting in apoptosis (Amaral et al., 2010). Considering tumor development, it has been shown that curcumin inhibits COX-2 synthesis through the AMPK pathway, which induces apoptosis in tumor progression (Lee YK. et al., 2009). In the NF-κB pathway, it exerts different effects on cells with different diseases. For example, in NAFLD and LF, it inhibits inflammation and protects hepatocytes from apoptosis through the inhibition of the NF-κB pathway. In contrast, in HCC, it promotes apoptosis in cancer cells by inhibiting the NF-κB pathway and promoting the transcription of apoptosis-related genes.

### 4.3 Limitation

1) The effectiveness of curcumin in the treatment of NAFLD, LF, and HCC has been determined by multiple pharmacodynamic index analyses and pathological examinations. However, since the therapeutic mechanisms of curcumin for these three diseases are diverse, further experiments are needed to explore the specific mechanisms of curcumin in systemic liver diseases. 2) In addition, multiple modes of administration, including gavage administration, intraperitoneal injection, and tail vein injection, were used in the studies of curcumin for the treatment of HCC in the literature. Therefore, this has led to a great heterogeneity in the dosage of curcumin administration. 3) The results of the meta-analysis showed a high degree of heterogeneity. This heterogeneity was influenced by various factors such as the duration of administration, dose administered, and delivery method. 4) Fifty-two articles were included in this study, but none of the studies described the investigators as being blinded during the experiment, which may have led to potential selection and implementation bias.

### 4.4 Implication

Based on the results of the quantitative analysis evaluated in this system, we can conclude that curcumin is applicable to a spectrum of NAFLD–LF–HCC models and that most liver diseases proceed based on the hepatic inflammatory microenvironment. In the development of a range of liver diseases, one of the most remarkable molecular changes driving the NAFLD–LF–HCC axis is the NF-κB signaling pathway, so anti-inflammatory processes play a crucial role in all three disease stages. More attention should be paid to the anti-inflammatory effects of curcumin. In addition to this, the poor bioavailability of curcumin can be improved in the following ways: 1. using adjuvants that interfere with the glucuronidation of curcumin; 2. combining curcumin with nanoparticle technology; 3. using liposomal curcumin or curcumin phospholipid complexes.

## 5 Conclusion

Curcumin has been shown to be effective in inhibiting the progression of NAFLD–LF–HCC at doses of 100–400 mg/kg over a 4–8 weeks duration with significant hepatoprotective effects, and its therapeutic mechanisms are related to multiple pathways, including anti-inflammatory, antioxidant, and apoptotic regulations, such as TLR4/NF-κB, Keap1/Nrf2, Bax/bcl2/Caspase3, and TGF-β/Smad3 signaling pathways, which are regulated in all stages of liver disease.

## Data Availability

The raw data supporting the conclusion of this article will be made available by the authors, without undue reservation.
