# Supplementary material for: Protective role of curcumin in disease progression from non-alcoholic fatty liver disease to hepatocellular carcinoma: a meta-analysis
Source: Front Pharmacol. 2024 Jan 19;15:1343193. doi: 10.3389/fphar.2024.1343193 (PMC10834658; doi:10.3389/fphar.2024.1343193)
Supplement: Supplementary file 1 [file Table1.DOCX]

***Supplementary Table 1. Search strategy in PubMed***

| **Search strategy** | | |
| --- | --- | --- |
| NAFLD | #1 | (Curcumin[Title/Abstract]) AND (curcumin[MeSH Terms]) |
|  | #2 | (fatty liver[Title/Abstract])) AND (fatty liver[MeSH Terms])) AND (NAFLD[MeSH Terms])) AND (NASH[MeSH Terms])) AND (non-alcoholic fatty liver disease[MeSH Terms]) |
|  | #3 | #1 and #2 |
| Liver fibrosis | #4 | (Curcumin[Title/Abstract]) AND (curcumin[MeSH Terms]) |
|  | #5 | (liver fibrosis[Title/Abstract])) AND (liver fibrosis[MeSH Terms])) AND (hepatic fibrosis[MeSH Terms])) |
|  | #6 | #4 and #5 |
| Liver cancer | #7 | (Curcumin[Title/Abstract]) AND (curcumin[MeSH Terms]) |
|  | #8 | (liver cancer[Title/Abstract])) AND (liver cancer [MeSH Terms])) AND (hepatocellular carcinoma[MeSH Terms])) AND (HCC[MeSH Terms])) |
|  | #9 | #7 and #8 |

| ***Supplementary Table 2. Possible mechanisms of Curcumin*** | | | |
| --- | --- | --- | --- |
| ***Studies*** | ***Category*** | ***Proposed mechanisms*** | ***Targets/pathways*** |
| Lee.et al 2022 | NAFLD | Curcumin phosphorylated AMPK inhibits the downstream target proteins C/EBP, PPARγ, and SREBP-1c through inhibiting preadipocyte differentiation, reducing liver TG levels, and thus ameliorating steatosis. Curcuminoids suppressing the TLR-2 and TLR-4 to inhibit NF-κB and MAPK signal pathways, and reduce inflammatory cytokines. | Upregulation: AMPK,  Downregulation: SREBP-1c, FAS, PPARγ, C/EBP, TLR-2, TLR-4 |
| Chen.et al 2022 | NAFLD | Curcumin alleviates hepatic steatosis, by inhibiting 11β-HSD1, which activates the AMPK/SIRT1 signaling pathway to regulates ACC- CPT-1and SREBP1- FASN. | Upregulation: SIRT1, AMPK, CPT-1  Downregulation: 11β-HSD1, SREBP1 |
| Elrazek.et al 2022 | NAFLD | Curcumin enhances lipolysis and β-oxidation by up-regulating the expression of lipases such as adipose triglyceride lipase, hormone-sensitive lipase, adiponectin, and AMP-activated protein kinase. | Upregulation: AMPK, CPT1  Downregulation: MDA, ROS |
| El-Hameed.et al 2021 | NAFLD | Nrf2 influences the changes in gene expression to reduce lipogenesis, ER stress, inflammation, oxidative stress, and fibrosis. | Upregulation: Nrf2  Downregulation: |
| Tong.et al 2021 | NAFLD | Curcumin inhibited the activation of macrophages to M1 macrophages and then reduce the expression of IL-1 β and TNF-α to alleviate hepatic dysfunction and inflammation during NASH | Upregulation:  Downregulation: the number of M1 macrophages |
| Sun.et al 2021 | NALFD | Curcumin rectified the dysregulated expression of SLC13A5/ACLY possibly via the AMPK−mTOR signaling pathway. inhibited both citrate transport and metabolism mediated by SLC13A5 and ACLY, respectively. These findings confirm that curcumin improves the lipid accumulation in the liver by blocking citrate disposition. | Upregulation: AMPK  Downregulation: SLC13A5, ACLY, acetyl-CoA,  mTOR |
| Li.et al 2021 | NAFLD | CUR is effective in modulating liver inflammation, improving intestinal integrity, controlling weight gain and serum lipid metabolism, and supporting a healthy structure of gut microbiota. | Upregulation:  Downregulation: |
| Lee.et al 2020 | NAFLD | Cur ameliorates NAFLD and insulin resistance by blocking hepatic lipogenesis and TG accumulation via the regulation of lipogenic and autophagy-related genes and inhibiting cellular apoptosis | Upregulation: AMPK  Downregulation: SREBP1, PPAR-γ, FAS |
| Feng.et al 2019 | NAFLD | Curcumin protects against HFD-induced hepatic steatosis by improving intestinal barrier function and reducing endotoxin and liver TLR4/NF-κB inflammation | Upregulation:  Downregulation: TLR4, NF-κB p65 nuclear translocation |
| Gheibi.et al 2019 | NAFLD | Cur ameliorates NAFLD-induced apoptosis, fatty degeneration and liver injury via up-regulating anti-oxidative responses by the action of GSH-Px and SOD and elevating TAC level, stabilizing mitochondrial membrane integrity by up-regulating bcl-2 and controlling p53 and caspase III expression/synthesis along with anti-inflammatory response by suppressing the production of iNOS, NO, SGPT, SGOT. | Upregulation: bcl-2  Downregulation: p53 and caspase III |
| Yan.et al 2018 | NAFLD | Nrf2/FXR/LXRα pathway might synergistically regulate both endogenous and exogenous metabolism in NAFLD mice and LXRα may be a novel therapeutic target of curcumin. | Upregulation: CYP3A, CYP7A, HNF-4α, Nrf2, FXR  Downregulation: CD36, SREBP-1c, FAS, LXRα |
| Cunningham.et al 2018 | NAFLD | Curcumin reduce antioxidant through NRF2, and reduce ACC to promote β-oxidation, And through the modulation of fibroblast growth factor 21 (FGF-21) via facilitation of fatty acid oxidation and gluconeogenesis. | Upregulation: Nrf2,  Downregulation: ACC, FAS, CD36, FGF-21 |
| Li.et al 2018 | NAFLD | Curcumin significantly reversed PPAR-α DNA hypermethylation status in the steatosis hepatocyte model. | Upregulation: PPAR-α  Downregulation: DNA hypermethylation |
| Ding.et al 2018 | NAFLD | Curcumin, as well as allopurinol, notably up-regulated miR-200a expression, accordingly, down-regulated TXNIP and inhibited NLRP3 inflammasome activation in fructose-fed rat livers and fructose-exposed BRL-3 A and HepG2 cells. | Upregulation: miR-200a expression  Downregulation: NLRP3, ASC, TXNIP |
| Feng.et al 2018 | NAFLD | Curcumin enhanced effects through improving oxidative stress, hepatic inflammation and lipid metabolism. | Upregulation:  Downregulation: SREBP-1c, pERK, TNF-α, pJNK |
| Mahmoud.et al 2018 | NAFLD | Cur inhibited NF-κB and reduce TNF-α to increase insulin sensitivity to increase glucose uptake in both visceral and subcutaneous adipocytes. | Upregulation:  Downregulation: NF-κB, TNF-α, IL-6 |
| Liu.et al 2017 | NAFLD | Cur suppressed the HFD-induced upregulated expression of CD36 and hepatic peroxisome proliferator activated receptor-𝛾 (PPAR-𝛾), a positive regulator of CD36. Cur dramatically activated cAMP response element-binding (CREB) protein, which negatively controls hepatic PPAR-𝛾 expression, via the activation of CREB inhibition of the hepatic PPAR-𝛾/CD36 pathway. | Upregulation: PPAR-α, CPT1  Downregulation: SREBP-1c, ACC, FAS, CD36, |
| Kim.et al 2016 | NAFLD | Cur through AMPK shutting down the anabolic pathway and promoting catabolism by upregulating PPARα and downregulating the activity of key lipid metabolic enzymes, such as, SREBP-1c, C/EBPα, and FAS. Consequently, curcumin suppressed fat accumulation in the liver and reduce formation of a fatty liver. | Upregulation: AMPK, PPARα  Downregulation: SREBP1, PPAR-γ, FAS, C/EBP |
| Wang.et al 2013 | NAFLD | Curcumin may inhibit the inflammatory response in the ‘second hit’ stage through the PPARγ/NF-κB pathway to suppressed TNF-alpha and IL-6, to regulating adipokines | Upregulation:  Downregulation: PPAR-γ, NF-Κb, TNF-α and IL-6 |
| Kuo.et al 2012 | NAFLD | curcumin activates STAT3 signaling and inhibits hepatic lipogenesis. the anti-inflammatory effect of curcumin is an integral part of the improvement of mitochondrial function in hepatic steatosis. Reversed mitochondrial dysfunction. | Upregulation: STAT3  Downregulation: SREBP-1c, FAS, ACC, NF-κB |
| Elswefy.et al 2020 | Liver fibrosis | Curcumin mediated through their anti-inflammatory and anti-apoptotic properties. | Upregulation: Bcl2,  Downregulation: caspase-3 |
| Gowifel.et al 2020 | Liver fibrosis | Curcumin exerted a significant upregulation of Nrf2/HO-1 gene expressions along with significant downregulation of NF-ĸB, TGF-β, and phospho-Smad3 protein expressions, as well as α-SMA and collagen-1 gene expressions. modulate Nrf2/HO-1, NF-κB, and TGF-β/Smad3 signaling pathways. | Upregulation: Nrf2, HO-1,  Downregulation: NF-κB, TGF-β, iNOS, Smad3 |
| Kong.et al 2020 | Liver fibrosis | Curcumin inhibits levels of reactive oxygen species (ROS) and oxidative stress in hepatocytes by activating PPAR-α, and regulates upstream signaling pathways of autophagy AMPK and PI3K/ AKT/mTOR, leading to an increase of the autophagic flow in hepatocytes. curcumin effectively reduced the occurrence of EMT in hepatocytes via blocking the TTC3/SMURF2/ SMADs axis and inhibited production of the extracellular matrix (ECM) by activating autophagy | Upregulation: LC3, PPAR-α, AMPK,  Downregulation: caspase-3 |
| Aquino.et al 2020 | Liver fibrosis | Curcumin decreased JNK and Smad3 phosphorylation. Furthermore, curcumin treatment decreased α-SMA and Smad3 protein and mRNA levels. Curcumin normalized GSH, and NF-κB, JNK-Smad3, and TGF-β -Smad3 pathways, leading to a decrement in activated hepatic stellate cells, thereby producing its antifibrotic effects by downregulating canonical and non-canonical Smad pathways, restoring Smad 7 levels, blocking NF-κB proinflammatory cytokine production, and decreasing the number of activated HSCs in the hepatic parenchyma | Upregulation:  Downregulation: JNK, Smad3, NF-κB |
| Khodarahmi.et al 2020 | Liver fibrosis | Demonstrated that increment of antioxidant defense of BDL rats by curcumin treatment correlates well with increase in PON1 activities via increase in PON1 gene and expression of protein and the regulatory genes contributed to the PON1gene in liver fibrosis. | Upregulation: PON1 protein  Downregulation: |
| Abo-Zaid.et al 2020 | Liver fibrosis | It enhanced the immune system response against fibrogenesis by suppressing pro-inflammatory cytokines such as TNF-α, IL-6, and TGF-β. | Upregulation:  Downregulation: TNF-α, IL-6, TGF-β |
| Eshaghian.et al 2018 | Liver fibrosis | curcumin treatment hepatic IR through down-regulation of SOCS3 and STAT3 and also up-regulation of IRS1. Furthermore, curcumin reduced inflammatory cytokines (IL-1β, TNF-α, iNOS, NF-κB and leptin), as well as oxidative stress, and subsequently ROS generation via down-regulation of Rac1, Rac1-GTP and NOX1, which in turn leads to attenuation of liver fibrosis through reduction of ERK1, HIF-1α and Sp1 expression levels. | Upregulation: IRS1  Downregulation: SOCS3, STAT3, RK1, HIF-1α and Sp1 |
| Qin.et al 2018 | Liver fibrosis | Curcumin may inhibit CXCL12/CXCR4 biological axis so that downstream RhoA/ROCK pathway is downregulated, and thus activation and migration ability of HSCs is suppressed. Therefore, curcumin could attenuate liver fibrosis by inhibiting CXCL12/CXCR4/RhoA axis. | Upregulation:  Downregulation: CXCL12, CXCR4 |
| Zhao.et al 2018 | Liver fibrosis | curcumin can reduce chemokines secretion by inhibiting KCs activation to decrease Ly6Chi monocyte infiltration in the treatment of liver fibrosis. | Upregulation:  Downregulation: CCL7, MCP-1 |
| Jin.et al 2016 | Liver fibrosis | Curcumin regulated senescence by inducting PPARγ/P53 pathway in activated HSCs, inhibiting fibrogenic properties in activated HSCs by curcumin. | Upregulation: Hmga1, PPARγ, P53  Downregulation: |
| Huang.et al 2016 | Liver fibrosis | Curcumin in inhibition of hepatic NF-κB-mediated inflammation in CCl4-induced liver fibrosis. | Upregulation:  Downregulation: JNK/, NF-κB |
| Peng.et al 2016 | Liver fibrosis | CUR treatment reversed liver injury *in vivo* and *in vitro*, possibly through down regulation of DNMT1, *α*-SMA, and Col1*α*1 and by demethylation of the key genes. | Upregulation:  Downregulation: DNMT1, α-SMA, Col1α1 |
| Zhang.et al 2015 | Liver fibrosis | Curcumin protected the rat liver from CCl4-caused injury and fibrogenesis in vivo by improving hepatic function, reducing ECM expression and inhibiting HSC activation. | Upregulation:  Downregulation: ECM expression |
| Swefy.et al 2015 | Liver fibrosis | Curcumin downregulated cyclooxygenase-2 (COX-2) and both mRNA and protein levels of nuclear factor kappa B (NF-κB) in fibrotic liver curcumin reduced the expression of CB1 in fibrotic liver. Curcumin alleviates hepatic fibrosis and dysfunction directly through inhibition of oxidative stress and inflammation. | Upregulation: Bcl2  Downregulation: NF-κB, CB1 |
| Zhao.et al 2014 | Liver fibrosis | Curcumin Protects against CCl4-Induced Liver Fibrosis in Rats by Inhibiting HIF-1α Through an ERK-Dependent Pathway. | Upregulation:  Downregulation: HIF-1α, p-ERK |
| Wang.et al 2012 | Liver fibrosis | Curcumin promotes TAA damaged cells to undergo apoptosis rather than necrosis by upregulating Bax and down-regulating Bcl-2 and Bcl-xL mRNA expression levels in vivo. Promotion of apoptosis not only decreases the number of necrotic cells during chronic liver injury, which inhibits the activation of inflammatory responses, but also prevents proliferation of hepatocytes containing damaged DNA that could progress to hepatocellular carcinoma. | Upregulation: P53, Bax  Downregulation: Bcl-2, |
| Yao.et al 2012 | Liver fibrosis | Curcumin significantly attenuated the severity of CCl4-induced liver inflammation and fibrosis through inhibition of TGF-β1/Smad signaling pathway and CTGF expression. | Upregulation: Smad7  Downregulation: TGF-β1, Smad2 and Smad3 |
| Bassiouny.et al 2011 | Liver fibrosis | Curcumin attenuated oxidative stress by increasing the content of hepatic glutathione within normal values, leading to the reduction in the level of lipid hydroperoxide. Curcumin remarkably suppressed inflammation by reducing levels of inflammatory cytokines, including TNF-α, NF-κB and IL-6. It also inhibited HSCs activation by elevating the level of PPARγ and reducing the abundance of TGF-β. | Upregulation: APE1, p53, PPARγ  Downregulation: TGF-β，TNF-α |
| Fu.et al 2007 | Liver fibrosis | curcumin protects the rat liver from CCl4-caused injury and fibrogenesis by suppressing hepatic inflammation, attenuating hepatic oxidative stress and inhibiting HSC activation. | Upregulation: PPARγ  Downregulation: PDGF, TGF-β |
