# Supplementary material for: Protective role of curcumin in disease progression from non-alcoholic fatty liver disease to hepatocellular carcinoma: a meta-analysis
Source: Front Pharmacol. 2024 Jan 19;15:1343193. doi: 10.3389/fphar.2024.1343193 (PMC10834658; doi:10.3389/fphar.2024.1343193)
Supplement: Supplementary file 2 [file Table2.DOCX]

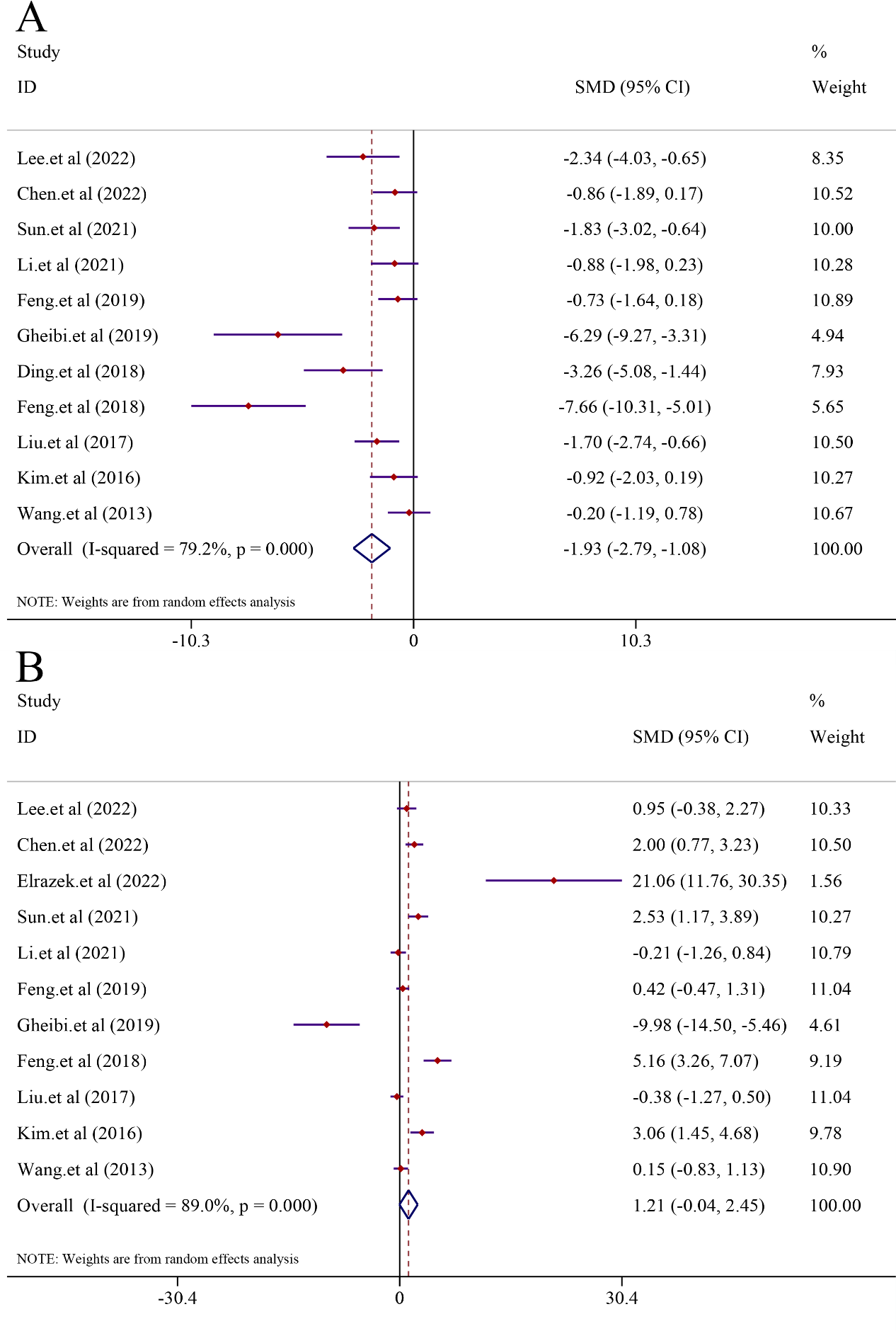
 Supplementary Figure 1. Effect of curcumin on LDL and HDL levels in NAFLD

(A. Pooled effect of HDL. B. Pooled effect of LDL)


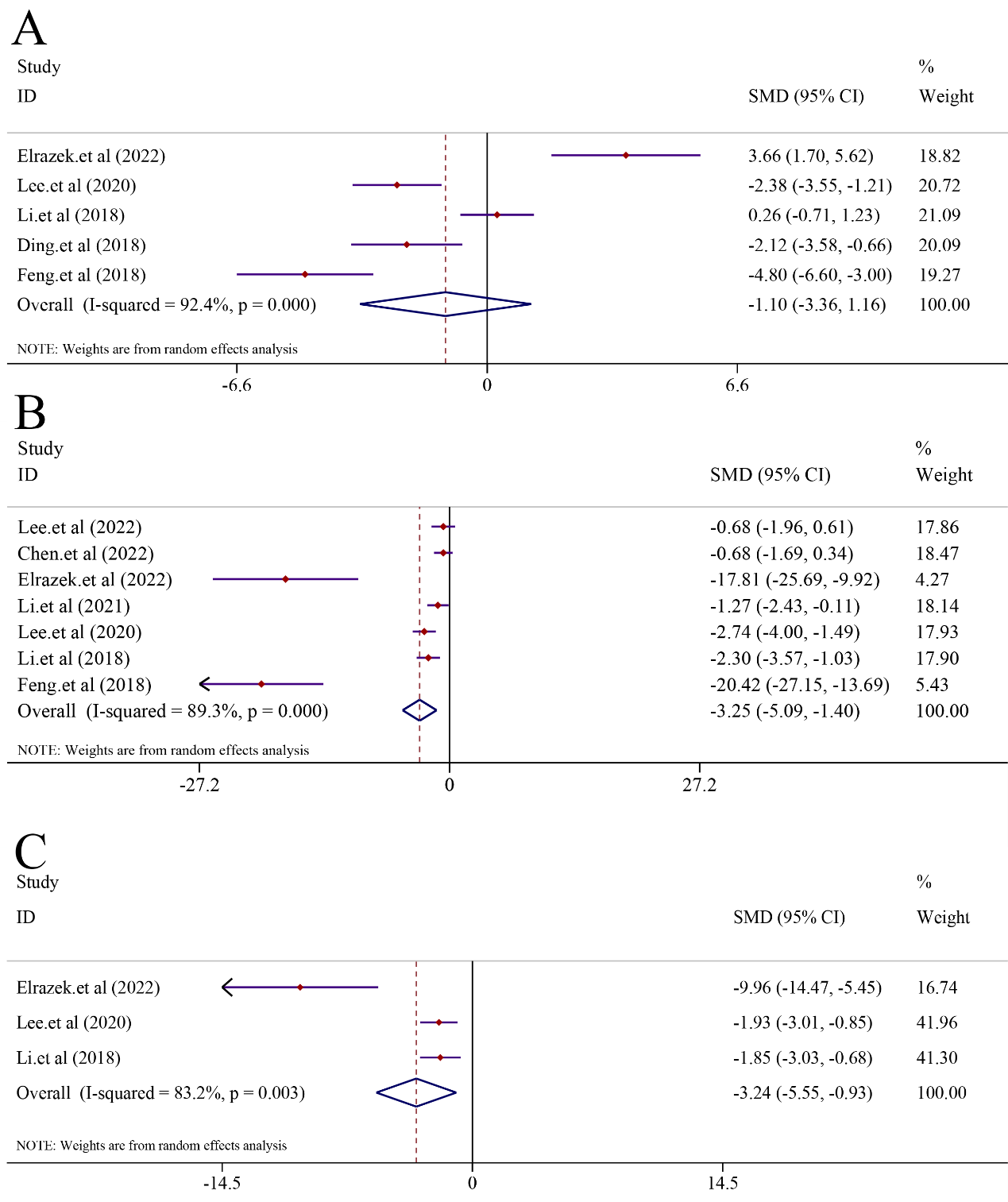


Supplementary Figure 2. Effect of curcumin on Insulin, Glucose and HOMA-IR levels in NAFLD

(A. Pooled effect of Insulin. B. Pooled effect of Glucose. C. Pooled effect of HOMA-IR.)


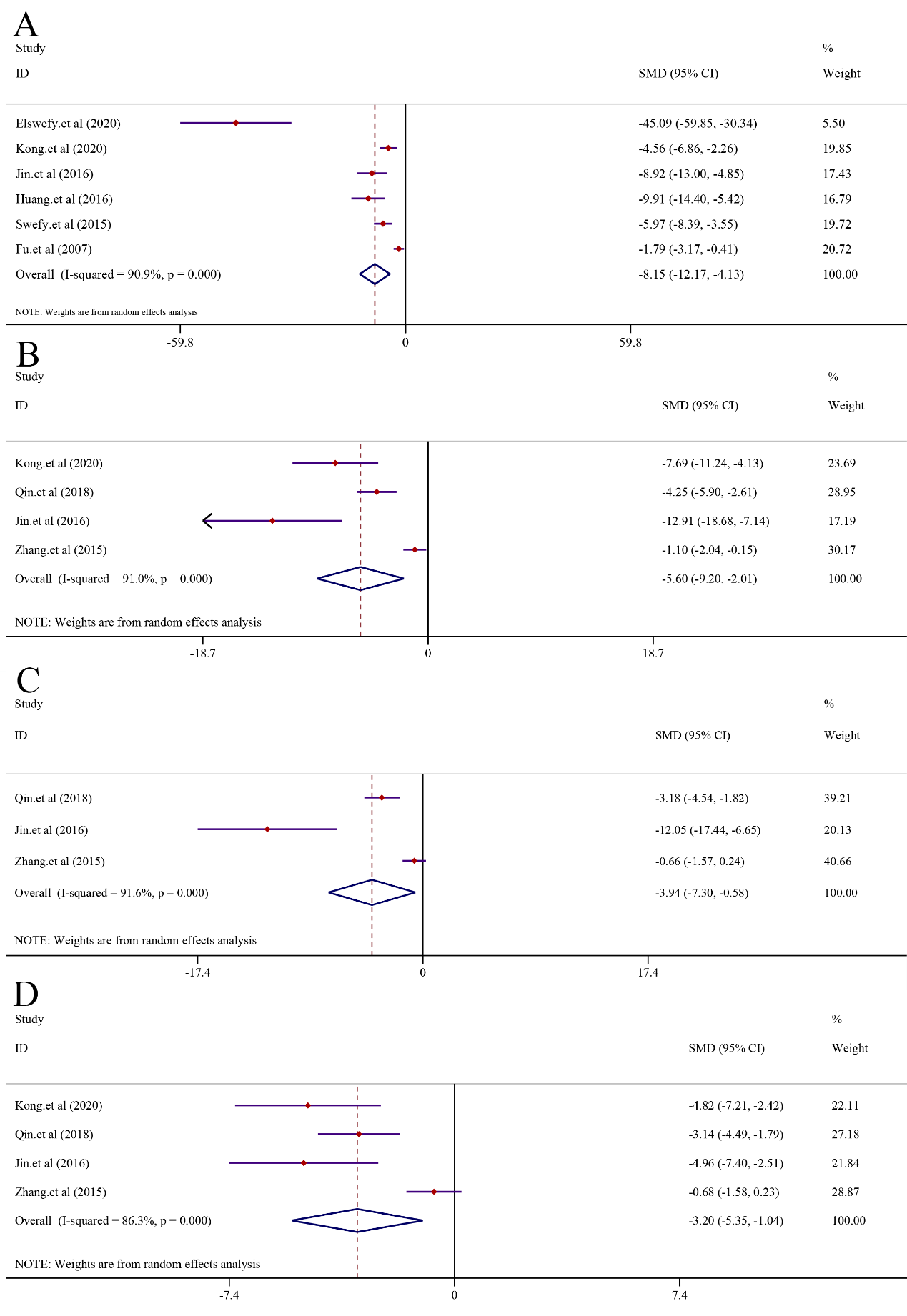
 Supplementary Figure 3. Effect of curcumin on Hydroxyproline, Hyaluronidase, Laminin and Procollagen Ⅲ in liver fibrosis

1. Pooled effect of Hydroxyproline. B. Pooled effect of Hyaluronidase. C. Pooled effect of Laminin. D. Pooled effect of Procollagen Ⅲ


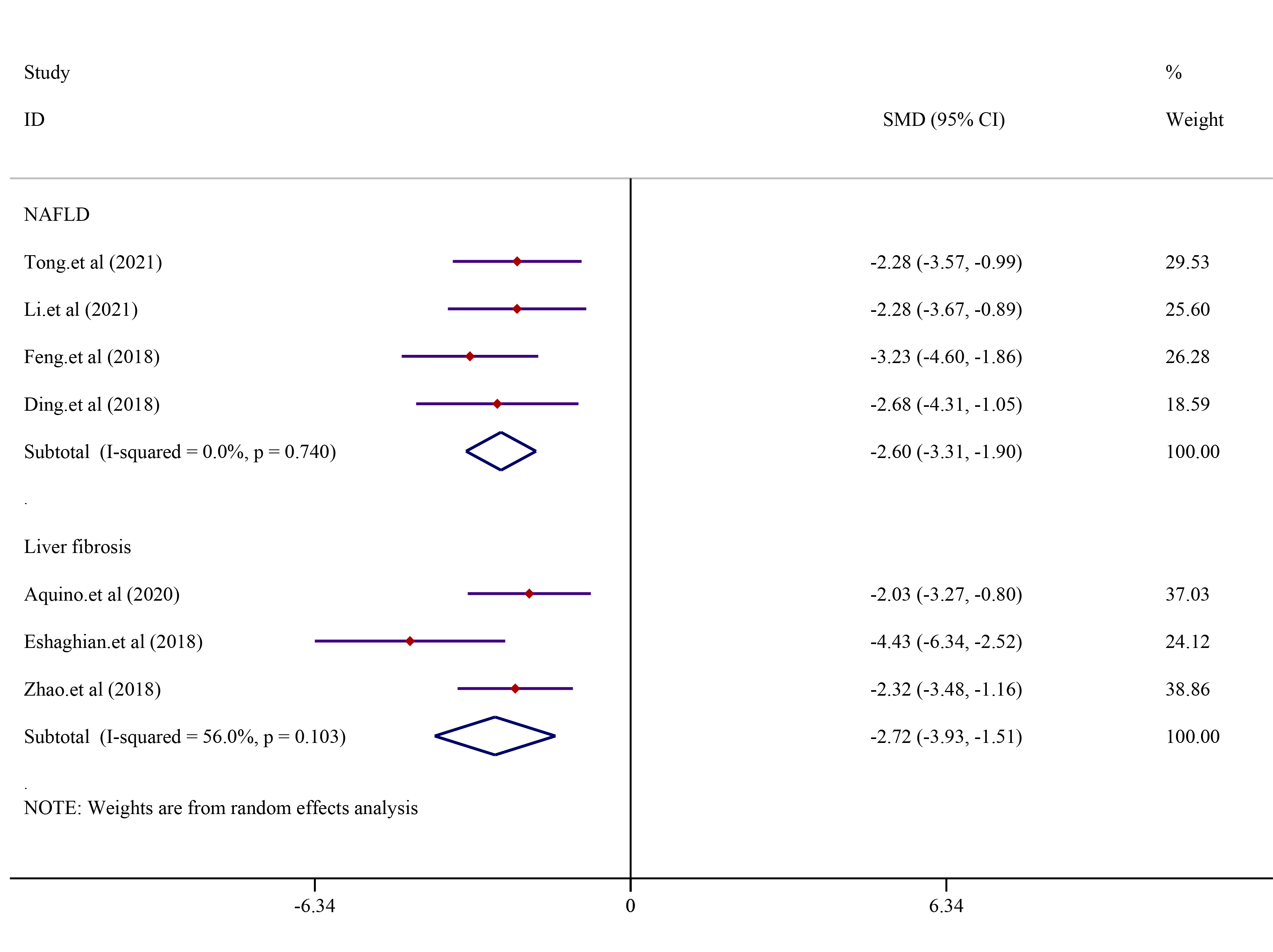


Supplementary Figure 4. Effect of curcumin on IL-1β levels.


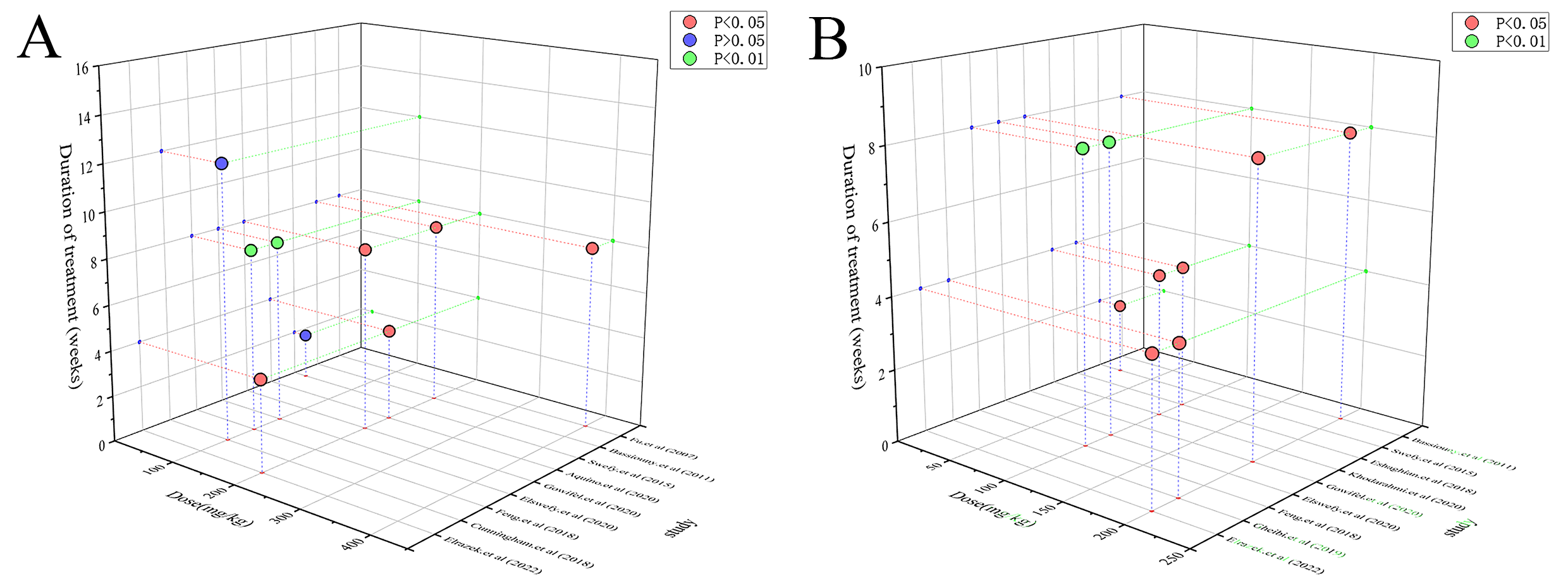


Supplementary Figure 5. 3D dose/time-effect images. (A)GSH; (B)MDA


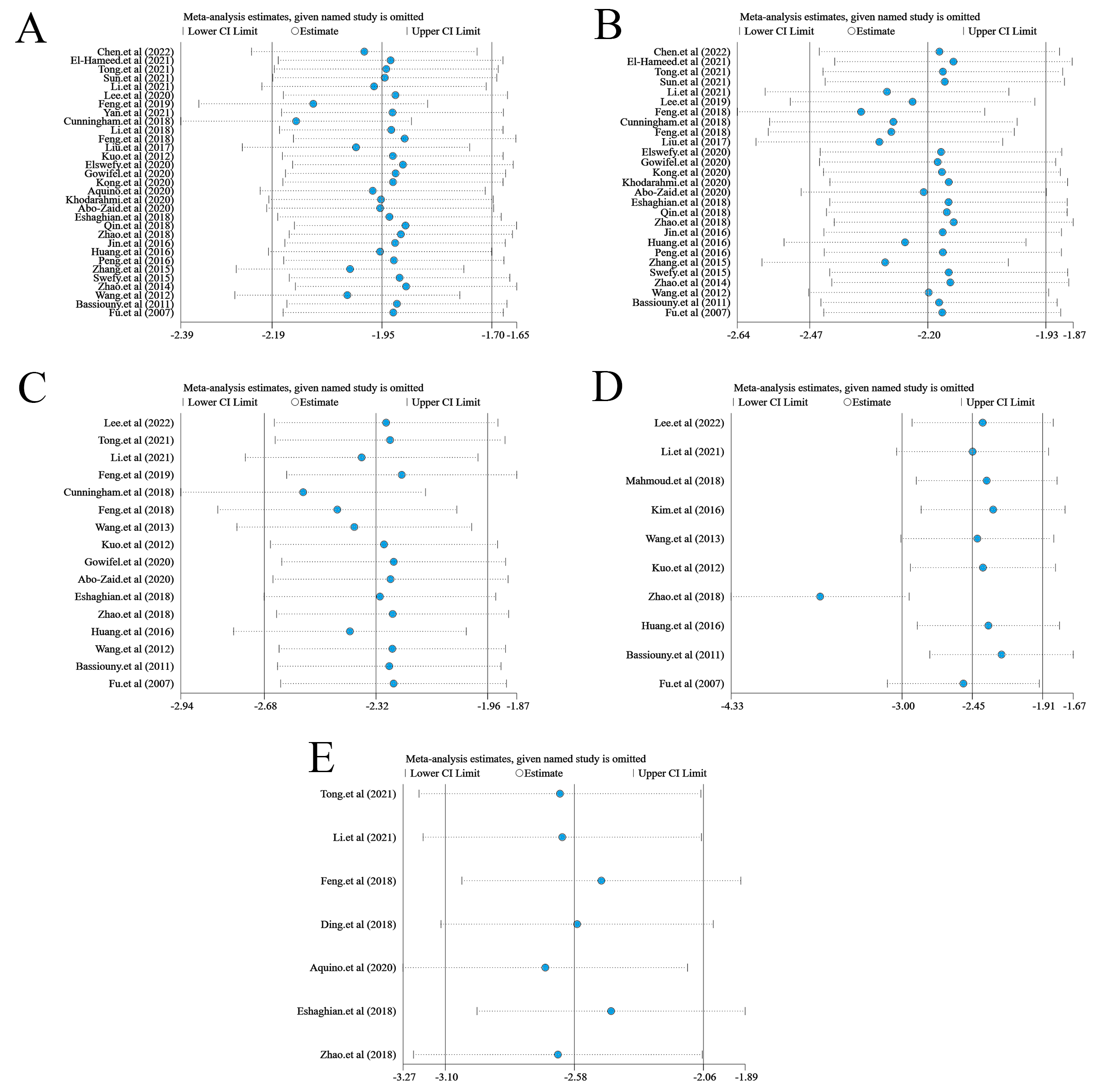


Supplementary Figure 6. The plot of Sensitivity test. (A) ALT; (B) AST; (C) TNF-α; (D) IL-6;

1. IL-1β.


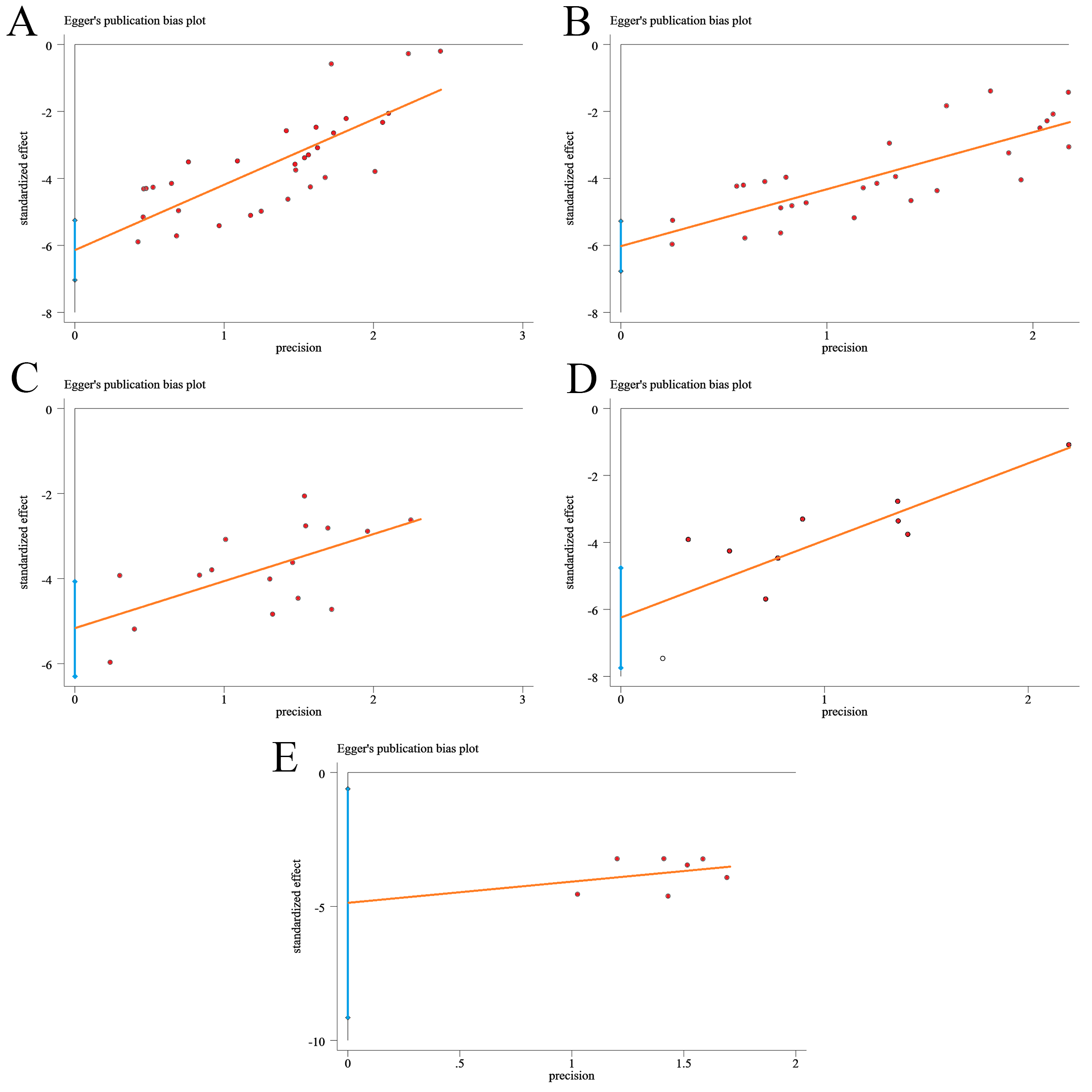


Supplementary Figure 7. Egger’s publication bias plot. (A) ALT; (B) AST; (C) TNF-α; (D) IL-6;

1. IL-1β.
